# Multi-USV Adaptive Exploration Using Kernel Information and Residual Variance

**DOI:** 10.3389/frobt.2021.572243

**Published:** 2021-05-28

**Authors:** Rajat Mishra, Teong Beng Koay, Mandar Chitre, Sanjay Swarup

**Affiliations:** ^1^Acoustic Research Laboratory, Tropical Marine Science Institute, National University of Singapore, Singapore, Singapore; ^2^NUS Environmental Research Institute, National University of Singapore, Singapore, Singapore; ^3^Department of Electrical & Computer Engineering, Faculty of Engineering, National University of Singapore, Singapore, Singapore; ^4^Singapore Centre for Environmental Life Sciences Engineering, Singapore, Singapore; ^5^Department of Biological Sciences, Faculty of Science, National University of Singapore, Singapore, Singapore

**Keywords:** multi-robot systems, informative path planning, Gaussian process, field validated, sampling hotspots, freshwater analysis

## Abstract

Using a team of robots for estimating scalar environmental fields is an emerging approach. The aim of such an approach is to reduce the mission time for collecting informative data as compared to a single robot. However, increasing the number of robots requires coordination and efficient use of the mission time to provide a good approximation of the scalar field. We suggest an online multi-robot framework *m-AdaPP* to handle this coordination. We test our framework for estimating a scalar environmental field with no prior information and benchmark the performance via field experiments against conventional approaches such as lawn mower patterns. We demonstrated that our framework is capable of handling a team of robots for estimating a scalar field and outperforms conventional approaches used for approximating water quality parameters. The suggested framework can be used for estimating other scalar functions such as air temperature or vegetative index using land or aerial robots as well. Finally, we show an example use case of our adaptive algorithm in a scientific study for understanding micro-level interactions.

## 1. Frameworks for Environmental Monitoring

### 1.1. Current Practices in Environmental Monitoring

Environmental processes often exhibit large scale features, generally in the range of kilometers, and vary both spatially and temporally. In order to monitor these processes through environmental parameters such as pH or dissolved oxygen (DO), it is ideal to have multi-fold coverage of the survey area. Buoys and floats equipped with environmental sensors are used to monitor water quality across different water resources such as oceans and freshwater systems. One of the widely used platforms is Argo Floats (Roemmich et al., 1999), which has helped in various scientific studies (Siswanto et al., 2008; Hosoda et al., 2009; Mignot et al., 2014; Stanev et al., 2014). A common approach is to place static buoys based on prior information from environmental modeling (Krause et al., 2008; Hart and Murray, 2010). Such an approach provides good temporal resolution, however, it is resource intensive as each buoy requires environmental sensors and regular maintenance.

**Figure 1:**
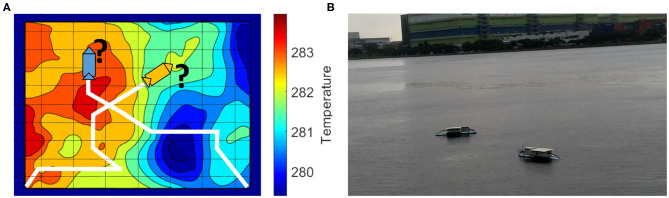
**(A)** A multi-robot scenario similar to the transect sampling task presented in Cao et al. (2013). The environmental field here is the sea surface temperature of an area in the Sea of Japan on January 21, 2018, taken from MUR SST dataset (JPL MUR MEaSUREs Project, 2010). **(B)** Our robots deployed in a local reservoir to perform adaptive monitoring to estimate dissolved oxygen in water.

More recently, robotic systems such as autonomous underwater vehicles (AUVs) and unmanned surface vehicles (USVs) are being increasingly used as fundamental data-gathering tools by scientists, catering to the need of environmental monitoring and sampling (Dunbabin and Marques, 2012). A large fraction of AUVs today are designed to carry out scientific data collection missions (Pascoal et al., 2000; Sukhatme et al., 2007; Zhang et al., 2012; Hitz et al., 2014; Koay et al., 2015). We also developed such robotic systems for water quality monitoring as shown in Figure 1B. Such robot-aided data collection has been also used to explain biological processes (Caron et al., 2008; Camilli et al., 2010). However, the use of these robots is still limited due to the complex spatio-temporal nature of the environmental parameters. Adaptive planning frameworks such as Informative Path Planning (IPP) are generally used to overcome such limitations and perform environmental monitoring missions (Zhang and Sukhatme, 2007; Smith et al., 2011; Cao et al., 2013; Hitz et al., 2017).

One of the challenges in using adaptive planning frameworks is the data collection process. In general, the IPP framework mitigates this challenge by evaluating paths using an informative criterion for unobserved locations (Sukhatme et al., 2007; Low et al., 2008, 2011; Yu et al., 2016; Ma et al., 2017), shown as an illustration in Figure 1A. The robot then traverses the path that provides maximum information as per the predefined criterion and collects data to give an estimate of the environment. In general, the IPP frameworks have three components: collecting data while traversing, adapting the robot's path to provide a good approximation of the field, and learning a model of the environmental field. The first component is self explanatory, whereas, the last two components are the key characteristics that define the behavior of all IPP frameworks.

The IPP frameworks generally plan the robot's path based on the data collected. Based on the frequency of this planning, the IPP frameworks can be classified as: non-adaptive algorithms (offline) which commit to a path and do not adjust based on new observations and adaptive algorithms (online) which alter the pre-planned paths on-the-fly based on the new observations. Several non-adaptive algorithms have been suggested in the past to solve near-optimal paths (Meliou et al., 2007; Hollinger and Singh, 2008; Singh et al., 2009; Das et al., 2010) using prior information of the field. However, the prior information for an environmental field may not be available for pre-planning of the robot's path. Such types of applications require the use of adaptive algorithms as the collection of information and path planning have to be synchronized.

The IPP frameworks can also be classified into multi-robot IPP frameworks (Singh et al., 2009; Low et al., 2011, 2012; Kemna et al., 2017) and single-robot frameworks (Zhang and Sukhatme, 2007; Hitz et al., 2017; Mishra et al., 2018). Each of these two classes of frameworks have their own advantages and disadvantages. The planning process for a single-robot framework is less complex compared to a multi-robot frameworks, however, covering large survey areas with a single robot may not be feasible due to limited resources. On the contrary, multi-robot frameworks can easily cover large survey areas by division-of-labor but this division-of-labor adds to the complexity of the multi-robot IPP framework. Moreover, multi-robot frameworks gather more data in a short amount of time and thus require methods that can estimate the field using large datasets in real-time. Such problems in model learning are currently not addressed and thus limits the usage to small datasets or small survey areas.

Another challenge in using adaptive algorithms is the online estimation of the survey field, as this estimate governs future waypoints in a robot's path. For example, in the case of water quality monitoring, a good approach may be to use off-the-shelf simulators like Delft3D (Deltares, 2006) or the Regional Oceanic Monitoring System (ROMS) (Moore et al., 2011). However, these simulators generally run on high performance clusters and such computational power is usually not available in robotic platforms. One good approach to combine these simulators with path planning is presented in Smith et al. (2010). In this approach, ROMS uses the data from various sensors to produce velocity profiles on a remote server, which can then be used by the robot for path planning. However, in areas where the sensors for ROMS are not present or the spatial resolution of ROMS's forecast is poor, such an approach will not work.

A commonly used approach in geostatistics (Le and Zidek, 2006; Webster and Oliver, 2007) is to assume that the spatio-temporal environmental field is realized from a probabilistic model called Gaussian processes (GPs). The computational power required for learning a Gaussian process model is comparatively much less than that required by physics-based simulators. Therefore, this approach has been commonly used in path planning (Sukhatme et al., 2007; Zhang and Sukhatme, 2007; Low et al., 2008, 2011, 2012). In Hitz et al. (2017), GPs and an information criterion were used to plan paths for an AUV to segment the environmental field into three different level sets. Similarly, using GPs, a path-planning algorithm based on entropy and information criterion is suggested in Cao et al. (2013). In all of these works, GP regression uses all the data collected during the survey. In a practical scenario, a water-quality sensor (YSI, 2017) can sense data at a frequency of about 1 Hz and thus running a robot with this sensor for an hour will provide about 3, 600 data points for learning the model. This means that the data collected during a survey can increase rapidly and therefore, the conventional method of doing GP regression may not be feasible. This problem can be solved by using sparse GPs.

An explanation of how sparse GPs can be integrated into a path planning framework is discussed in Ma et al. (2017). This recent work is directed toward long-term monitoring and overcomes the spatial and temporal changes by updating the GP model based on an information criterion. Although it is a good single-robot framework, the sparse GP point selection can be improved with more data-driven sparse GP variants such as sparse pseudo-inputs Gaussian processes (SPGP) (Snelson and Ghahramani, 2006). The combination of such sparse GP models and time-constrained mission planning for multi-robot frameworks is still lacking.

### 1.2. Practical Constraints in Using a Team of Robots

Our objective is to obtain a good approximation of a scalar environmental field, such as temperature, conductivity, or chlorophyll concentrations, using a team of robots within a fixed amount of time. We previously published a framework for estimating scalar fields using a single robot (Mishra et al., 2018). A common problem in using a single robot is the limitation on the area it can cover within a finite time, limiting the total collected information. Such problems with single robot scenarios can be easily resolved by using a team of robots to collect more information, however, these robots should be coordinated to collect the information efficiently.

An entropy-based method for multi-robot operation (Cao et al., 2013) generates a set of waypoints using dynamic programming. However, this framework only considers transect environmental fields, where robots can only move along one spatial direction and generate waypoints based on the assumption that fields are anisotropic. Another multi-robot framework uses a lawn mower to obtain preliminary information, and then a leader robot makes decisions to adapt the lawn mower pattern for the team of robots (Chen et al., 2012). Such an approach is helpful for adapting lawn mower patterns, however, following these straight paths consumes time and collects repetitive information. A similar approach is described in Petillo (2015), where the robots maintain a formation and adapt the formation to cover a larger area. Vehicles with motion constraints such as gliders can make use of these frameworks but robots that do not have such strict motion constraints may benefit from a more flexible planning framework.

We are interested in a multi-robot framework that can be used for a team of robots such as AUVs, impose fewer motion constraints, and finish the monitoring task within a fixed amount of time. Moreover, an important component missing in the multi-robot frameworks is the computation time for making decisions. The computation time can be ignored in cases where it is insignificant compared to the overall mission time. However, the framework's task is to finish collecting data within a short amount of time and thus computation time is an important component of our overall mission time. For example, if each decision iteration takes about 5 s to compute and iteration is repeated every 30 s, then during a mission of 600 s, decisions are taken about 20 times. In such a scenario, the computation time will consume more than 15% of the mission time and thus leave less time for data collection.

### 1.3. IPP Frameworks and Scientific Experiments

Adaptive monitoring frameworks are commonly used for estimating scalar environmental fields such as chlorophyll concentration and temperature. The examples for integrating these estimated fields into biological studies or the relevance for biological studies is still not well-established. Frameworks are designed for scientific experiments such as estimating hotspots or tracking a certain phenomenon, yet the process of using these estimated fields from a biological or geological standpoint is generally missing. This is especially true in the studies to understand the micro-level relationships between the estimated fields and the microorganisms living in it.

Scientists have attempted to establish the connection between the fields estimated using robots and various environmental phenomena. One such work tracks hydrocarbon plumes and bio-degradation at the Deepwater Horizon site (Newman et al., 2008; Camilli et al., 2010). This work focused on developing a framework to observe the bio-degradation of the hydrocarbon plume and it is a good example of tracking a biological phenomenon to understand it at a macro-scale. Another interesting approach for establishing scientific relevance is discussed in Das et al. (2015). In this approach, the authors designed two frameworks, one to make the sampling decisions, and another to estimate the concentration of a pathogen based on the sensor values. The focus of this work was to select samples from a predefined path and estimate the concentration of a particular pathogen.

We are interested in establishing a use case for our framework in identifying the micro-scale species associated with a water-quality parameter. The high concentration regions of these parameters can be both harmful and beneficial to the ecosystem, depending on the biological and chemical characteristics (Darrouzet-Nardi and Bowman, 2011; Zhu et al., 2013; Palta et al., 2014). It is important to find and sample these regions and discover the associated microbial communities. Sampling from hotspots of oxygen minimum zones has helped in understanding a microorganism's role in terrestrial nitrogen loss in inland waters (Zhu et al., 2015). Therefore, estimating the scalar fields and sampling from the hotspots of parameters such as DO is useful in understanding the environmental processes.

We introduce a multi-robot IPP framework *m-AdaPP* with constraints on mission time for estimating a scalar environmental field. Our aim is to coordinate a team of robots to get a good approximation of the scalar field and finish the overall mission in a fixed amount of time. We make use of a sparse GP method to provide an estimate of the field and the corresponding variance. The paths are evaluated to minimize the overall variance and we include the time taken for this evaluation in our overall mission time. We test the coordination and field estimation performance of our framework using a sea surface temperature dataset in simulation. We also examine the performance of our framework against two multi-robot IPP algorithms, a greedy algorithm and a distributed planning algorithm. We use an approach similar to that shown in Kemna et al. (2017) and the greedy benchmark algorithm as shown in Hitz et al. (2017) to simulate the greedy behavior. The two comparisons with a greedy and distributed algorithm will help us examine the performance gains when using a non-myopic and centralized planning approach. Finally, we compare our framework's performance against the conventional lawn mower patterns for estimating environmental fields, and show that our framework performs well. We also present an approach for integrating our framework into a scientific study.

## 2. Sparse Gaussian Processes

GP models are commonly used for non-parametric regression problems (Rasmussen and Williams, 2004), such as spatial data modeling (Stein, 2012), image thresholding (Oh and Lindquist, 1999), and soil modeling (Hengl et al., 2004). In a standard GP problem for spatial data regression, the training data set **D** consists of *N* vectors each composed of two elements $\text{X}={\left\{{\text{x}}_{n}\right\}}_{n=1}^{N}$ and corresponding target values $\text{y}={\left\{y_{n}\right\}}_{n=1}^{N}$ with a Gaussian measurement noise. The likelihood of observed values **y** can be given as $p\left(\text{y}|\text{f}\right)=N\left(\text{y}|\text{f},\sigma^{2}\text{I}\right)$ where **f** is the underlying latent function and σ^2^**I** is the noise term. Placing a zero mean prior and a covariance function given by $K\left({\text{x}}_{n},{\text{x}}_{n^{\prime }}\right)$ and parameterized by θ, the distribution for a new input **x** is given by:

(1) $$\begin{array}{r} p(y|x,D,\theta )=N(y|k_{x}^{\;⊤}{\left(K_{N}+\sigma^{2}I\right)}^{-1}y,K_{x,x}\\ -k_{x}^{\;⊤}{\left(K_{N}+\sigma^{2}I\right)}^{-1}k_{x}+\sigma^{2})\text{,} \end{array}$$

where ${\left[{\text{k}}_{x}\right]}_{n}=K\left({\text{x}}_{n},\text{x}\right)$, ${\left[{\text{K}}_{N}\right]}_{n,n^{\prime }}=K\left({\text{x}}_{n},{\text{x}}_{n^{\prime }}\right)$, and $K_{\text{x},\text{x}}=K\left(\text{x},\text{x}\right)$. As it can be observed from (1), the computation time for large datasets will be as high as the prediction, and even the training scales with *N*^3^ due to inversion of the covariance matrix, where *N* is the total number of datapoints. Sparse GPs overcome this problem by having sparse approximation of the full GP using only *M* points, where *M* ≪ *N*. In general, the selection of this subset of *M* points is based on information criteria (Seeger et al., 2003).

A common problem with information criterion-based sparse GP methods is the absence of a good method to learn the kernel hyperparameters, because the subset selection and hyperparameter optimization are generally done independently. Moreover, when using *automatic relevance determination* (MacKay, 1998) covariance function, learning bad hyperparameters can adversely affect the prediction performance. The SPGP framework solves this problem by constructing a GP regression model which finds the active subset and hyperparameters in one smooth joint optimization.

### 2.1. Sparse Pseudo-Input Gaussian Processes

In a standard GP model (Rasmussen and Williams, 2004) with zero mean prior, the kernel function is solely responsible for estimating the mapping between the input vector and the target values as shown in (1). Therefore, assuming the hyperparameters of the kernel function are known, the predictive function is effectively parameterized by **D**. In the case of SPGP, this parameterization is done using the pseudo data set $\bar{\text{D}}$ of size *M* ≪ *N*, which has pseudo-inputs $\bar{\text{X}}={\left\{{\bar{\text{x}}}_{m}\right\}}_{m=1}^{M}$ and corresponding pseudo targets $\bar{\text{f}}={\left\{{\bar{f}}_{m}\right\}}_{m=1}^{M}$. The pseudo targets are denoted as $\bar{f}$ instead of $\bar{y}$ because these targets do not represent the observed values and therefore, adding the noise variance σ^2^ can be omitted. The actual prediction distribution has the noise variance and is given as:

(2) $$\begin{array}{l} p(y|x,D,\theta )=N(y|k_{x}^{\;⊤}K_{M}^{-1}\bar{f},K_{x,x}-k_{x}^{\;⊤}K_{M}^{-1}k_{x}+\sigma^{2})\text{,} \end{array}$$

where ${\left[{\text{K}}_{M}\right]}_{mm^{\prime }}=\text{K}\left({\bar{\text{x}}}_{m},{\bar{\text{x}}}_{m^{\prime }}\right)$ and ${\left[{\text{k}}_{x}\right]}_{m}=K\left({\bar{\text{x}}}_{m},\text{x}\right)$, for m = 1, 2…, *M*. On comparing (1) and (2), one can clearly observe the reduced computation burden for the inversion of the covariance matrix, from a matrix **K**_*N*_ with *N* × *N* entries to a matrix **K**_*M*_ with *M* × *M* entries. Following the derivation in Snelson and Ghahramani (2006), the predictive distribution is given as a new input **x**_∗_ as:

(3) $$\begin{array}{r} p\left(y|{\text{x}}_{*},\text{D},\bar{\text{X}}\right)=N\left(y_{*}|\mu_{*},\sigma_{*}^{2}\right)\text{,} \end{array}$$

where

(4) $$\begin{array}{r} \mu_{*}=k_{*}^{\;T}Q_{M}^{-1}K_{MN}(\Omega +\sigma^{2}I)y \end{array}$$

(5) $$\begin{array}{r} \sigma_{*}^{2}=K_{*,*}-k_{*}^{⊤}(K_{M}^{-1}-Q_{M}^{-1})k_{*}+\sigma^{2}\text{.} \end{array}$$

The derivation of **Q**_*M*_ is omitted here for brevity but these are present in Snelson and Ghahramani (2006). The main cost in computing **Q**_*M*_ is the inversion of a diagonal matrix (Snelson and Ghahramani, 2006). Using the spatial data as input, μ_∗_ will represent the mean predicted field and the variance $\sigma_{*}^{2}$ will constitute the uncertainty in this prediction. The pseudo points $\bar{\text{X}}$, parameters θ, and noise variance σ^2^ are learned in one joint optimization given by (6). This joint optimization aims at learning a generative model by maximizing the marginal likelihood with respect to the pseudo points and kernel parameters.

(6) $$\begin{array}{r} p\left(\text{y}|\text{X},\theta ,\sigma^{2}\right)=N\left(0,{\text{K}}_{NM}{\text{K}}_{M}^{-1}{\text{K}}_{MN}+\Omega +\sigma^{2}\text{I}\right) \end{array}$$

We follow the suggestions given in Snelson and Ghahramani (2006) for initialization of *M* pseudo points and the kernel function and learn these parameters to get a representative model of the collected data. Moreover, the scalar environmental fields can be non stationary (Cao et al., 2013) and up to a certain extent, SPGP is capable of modeling non-stationary GP processes through its pseudo-inputs, which gives it an edge over other sparse GP methods.

## 3. Problem Formulation

We follow the common notations stated in Table 1 throughout our formulation and the suggested solution for consistency. Broadly, our problem statement is to find a path for a team of *H* robots and collect representative data to provide a good estimate of the environmental field and finish this task within a fixed amount of time *T*. This statement can be represented as:

(7) $$\begin{array}{r} \underset{{\hat{\text{P}}}_{t}\in {\hat{\Lambda }}_{t}}{\arg\min}\frac{1}{\left|\tilde{X}\right|}\underset{\tilde{X}}{\int }(Y\left(\text{x}\right)-\hat{Y}\left(\text{x},{\hat{\text{D}}}_{t}\cup {\hat{\text{D}}}_{T-t,{\hat{\text{P}}}_{t}}\right){)}^{2}d\text{x}\text{,} \end{array}$$

such that,

(8) $$\begin{array}{r} T\left({\hat{\text{P}}}_{t}\right)\leq T-t\text{, and} \end{array}$$

(9) $$\begin{array}{r} {\hat{\text{P}}}_{t}^{0}={\text{x}}_{t,1:H}\text{,} \end{array}$$

where ${\hat{\text{P}}}_{t}$ is a set containing one path for each robot and given as:

(10) $$\begin{array}{r} {\hat{\text{P}}}_{t}=\left\{{\text{P}}_{t,1},\;{\text{P}}_{t,2},\;{\text{P}}_{t,3},\text{…}{\text{P}}_{t,H}\right\}\text{,} \end{array}$$

and each of these paths {**P**_*t*, 1_, **P**_*t*, 2_, **P**_*t*, 3_, …**P**_*t, H*_} is a set of locations given by {**x**_*t*:*T*, 1_, **x**_*t*:*T*, 2_, **x**_*t*:*T*, 3_, …**x**_*t*:*T, H*_}. Similarly, ${\hat{\Lambda }}_{t}$ is a set containing all the paths for each robot and it is given as:

(11) $$\begin{array}{r} {\hat{\Lambda }}_{t}=\{\Lambda_{t,1},\;\Lambda_{t,2},\;\Lambda_{t,3},\ldots \Lambda_{t,H}\}\text{.} \end{array}$$

The function Y(·) in (7) is the field over the spatial domain $\tilde{X}$ and ${\hat{\text{D}}}_{t}$ is the data collected by all the robots and thus ${\hat{\text{D}}}_{t}=\left\{{\text{D}}_{t,1}\text{,}{\text{D}}_{t,2}\text{,}{\text{D}}_{t,3}\text{…}{\text{D}}_{t,H}\right\}$, where **D**_*t, i*_ is the data collected by robot *i* until time *t*. The function $\hat{Y}\left(\cdot ,\cdot \right)$ is the estimated function of the field at time *t* using the collected data ${\hat{\text{D}}}_{t}$ and the data yet to be collected ${\hat{\text{D}}}_{T-t,{\hat{\text{P}}}_{t}}$ by traversing paths given by ${\hat{\text{P}}}_{t}$. The path **P**_*t, i*_ and the set of collection of paths Λ_*t, i*_ in (10) and (11) represent the candidate paths for robot *i*. Moreover, ${\hat{\text{P}}}_{t}^{0}$ in (9) are the starting locations, which are also the locations of all the *H* robots at time *t*. All the paths in the set ${\hat{\Lambda }}_{t}$ start from the locations given by ${\hat{\text{P}}}_{t}^{0}$. Finally, the function T(·) provides an estimate of the time to traverse a path. In our problem statement, we have defined the measure of goodness as a low mean squared error over the complete spatial domain. The current form of the problem statement is not solvable as we cannot get the information about Y(·) without sampling or visiting locations and thus without actually traversing a set of paths ${\hat{\text{P}}}_{t}$, we cannot obtain the target values ${\text{y}}_{t:T}={\left\{y_{i}\right\}}_{i=t}^{T}$ for yet to be visited locations. To overcome this, we can make use of characteristics of a GP model to make problem (9) solvable. The function $\hat{Y}\left(\cdot ,\cdot \right)$ can be learned using a GP model and it can be written as $N\left(\mu_{*},\sigma_{*}^{2}\right)$, where μ_∗_ should represent a close approximation of Y(·) if the learned GP model is a good fit and the overall variance $\sigma_{*}^{2}$ is low. Therefore, we can re-write (7) as:

(12) $$\begin{array}{r} \underset{{\hat{\text{P}}}_{t}\in {\hat{\Lambda }}_{t}}{\arg\min}\frac{1}{\left|X\right|}\underset{X}{\sum }\sigma_{*}^{2}\left(\text{x},{\hat{\text{D}}}_{t},{\hat{\text{P}}}_{t}\right). \end{array}$$

It is important to take note of two changes between (7) and (12). First, we have replaced ${\hat{\text{D}}}_{T-t,{\hat{\text{P}}}_{t}}$ with just ${\hat{\text{P}}}_{t}$ as we can get an estimate of the variance without sensing the target values and only the spatial locations given by ${\hat{\text{P}}}_{t}$ are sufficient. However, the estimated variance depends on ${\hat{\text{D}}}_{T-t}$ and it will be updated using (5) whenever the robot collects more data ${\hat{\text{D}}}_{t}$. Therefore, our planning problem can be seen as collecting good data such that the overall variance becomes low.

**Table 1:** Description of all the commonly used notations in all the sections.

**Notation**	**Description**
$\tilde{X},X$	Continuous and discretized spatial domain.
*H*	Total number of robots.
**x**	2D location vector.
**x**_*t, i*_	Location of a robot *i* at time *t*.
**x**_*t*, 1:*H*_	Location of all the *H* robots at time *t*.
**x**_*t*:*T, i*_	Location of robot *i* from time *t* to *T*.
**x**_*t* + *T*_*s*_, 1:*H*_	Location of all the *H* robots at time *t* + *T*_*s*_.
**y**_*t*:*T*_	Measured scalar values by the team of robots between time *t* and *T*.
Y(·)	Scalar field over the continuous domain $\tilde{X}$.
${\hat{\text{D}}}_{t}$	Data collected from all the *H* robots till time *t*.
μ_∗_, $\sigma_{*}^{2}$	Estimated mean and variance by the sparse GP.
**c**_*t, i*_	Representative location of the cell at time *t*, visited by robot *i*.
**A**_**c**_*t* + *T*_*s*_, *i*__	Set of available actions at time *t* + *T*_*s*_ when a robot *i* visits a cell **c**_*t* + *T*_*s*_, *i*_.
*V*(**c**_*t, i*_)	Value function of a cell **c**_*t, i*_ for a robot *i* at time *t*.
π(**c**_*t, i*_)	Policy for the cell **c**_*t, i*_ at time *t*.
$R\left({\text{c}}_{t,i},a\right)$	Reward for taking action *a* when the robot *i* is in cell **c**_*t, i*_ at time *t*.
**P**_*t, i*_	A path for robot *i* for the remaining time of *T* − *t*.
$T\left({\text{P}}_{t,i}\right)$	Time for traversing a path **P**_*t, i*_ by robot *i* at time *t*.
${\text{P}}_{t,i}^{0}$	Starting location of the path for the robot *i* at time *t*.
Λ_*t, i*_	A set of all the paths **P**_*t, i*_ for a robot *i* for the remaining time *T* − *t*.
${\hat{\text{P}}}_{t}$	A set of paths for all the *H* robots for the remaining time *T* − *t*.
${\hat{\Lambda }}_{t}$	A set containing all the paths for all the robots for the remaining time *T* − *t*.
*T*_*s*_	Time interval for planning iterations.
Φ_*t* + *T*_*s*__	Set of all available actions for the team of robot at time *t* + *T*_*s*_.
$\varphi_{t+T_{s}}^{\prime }$	One set of actions for the team of robots at time *t* + *T*_*s*_.
$U\left(\varphi_{t+T_{s}}^{\prime }\right)$	Sum of cell variances reached by all the robots on taking the actions given by $\varphi_{t+T_{s}}^{\prime }$.
ϑ_*T* − (*t* + *T*_*s*_)_(·)	Represents the potential of reducing variance in the remaining time *T* − (*t* + *T*_*s*_).

Second, the problem statement given by (7) is in the continuous domain $\tilde{X}$. This means the number of paths in the set ${\hat{\Lambda }}_{t}$ will be large and searching for the optimal path ${\hat{\text{P}}}_{t}^{*}$ that satisfies our problem statement will be difficult. A common approach to reduce such complexity is to discretize the continuous domain $\tilde{X}$ into a grid X. In this scenario, each location **x** will generally have eight neighbors and thus for each location the decision will be to select which of these neighbors to visit. Finally, the constraints on (12) will be:

(13) $$\begin{array}{r} T\left({\text{P}}_{t,i}\right)+\tau \leq T-t\text{, and} \end{array}$$

(14) $$\begin{array}{r} {\text{P}}_{t}^{0}={\text{x}}_{t,i}\;\forall i\in \left[1,H\right]\text{,} \end{array}$$

where the new addition τ in comparison to (8) represents the computation time for each decision and **x**_*t, i*_ represents the location of robot *i* at time *t*. The constraints given by (13) represent that each robot will have less than *T* − *t* time available for collecting data. However, we can absorb τ inside $T\left({\text{P}}_{t,i}\right)$ if the computation can be done while traversing. This will require taking a decision for the next location while collecting data. The current formulation given by (12) will not allow this as the decision made at time *t* is possible only after collecting all the data ${\hat{\text{D}}}_{t}$ until time *t*. However, we can use the data ${\hat{\text{D}}}_{t}$ to make a decision for the next location **x**_*t* + 1_ and collect more data while traveling from **x**_*t*_ to **x**_*t* + 1_. This will change the problem statement to:

(15) $$\begin{array}{r} \underset{{\hat{\text{P}}}_{t}\in {\hat{\Lambda }}_{t+1}}{\arg\min}\frac{1}{\left|X\right|}\underset{X}{\sum }\sigma_{*}^{2}\left(\text{x},{\hat{\text{D}}}_{t},{\hat{\text{P}}}_{t}\right)\text{,} \end{array}$$

such that,

(16) $$\begin{array}{r} \tau \leq T\left(\left\{{\text{x}}_{t,i},{\text{x}}_{t+1,i}\right\}\right)\text{ and} \end{array}$$

(17) $$\begin{array}{r} T\left({\text{P}}_{t,i}\right)\leq T-t\text{ and} \end{array}$$

(18) $$\begin{array}{r} {\text{P}}_{t,i}^{0}={\text{x}}_{t+1,i}\;\forall i\in \left[1,H\right]\text{,} \end{array}$$

where ${\hat{\Lambda }}_{t+1}$ represents the set of all paths for each robot *i* from its next location **x**_*t* + 1, *i*_. This formulation changes (13) to (17) but introduces a new constraint given by (16), which suggests that computation time should be less than or equal to the time taken by the robots to travel to the next location. The set ${\hat{\Lambda }}_{t+1}$ can be visualized as a state space too. This state space will be a convolution of multiple state spaces given by {Λ_*t* + 1, *i*_} and its starting state given as **s**_*t*_ = {**x**_*t* + 1, *i*_} ∀*i* ∈ [1, *H*]. The state space of the set ${\hat{\Lambda }}_{t+1}$ at each planning iteration aims to reduce the variance and this is similar to the problem of selecting locations as described in Singh et al. (2009), which is shown to be an **NP**-hard search problem. Therefore, we need a framework to transform this search problem that can be solved in polynomial time and provide a good estimate of the environmental field.

## 4. Multi-Robot Planning Framework

We suggest a centralized framework, named as *m-AdaPP*, to efficiently search through the state space given by ${\hat{\Lambda }}_{t+1}$ and collect data using the kernel information to get a good estimate of our field. This algorithm follows the basic IPP framework and thus has the three components, which are planning, model learning, and collecting data. As discussed in the section before, we learn the model and plan for the next location while the robots are traveling and collecting more data. We make use of the spatial decomposition approach as explained in Mishra et al. (2018) and reduce our search space by discretizing the grid into cells.

There are three constraints on our planning as shown in our problem formulation. These are the limits on each robot's total mission time *T*, bounds on the computation time used for planning, and each robot's starting point. Although the planning is done over cells instead of locations, this does not mean that robots do not collect data while traveling from one cell to another. The data are collected as and when the sensors provide a scalar value of the field, defined by the sensor's frequency rate. These data are then stamped against the current location of the robot and sent to a central server. This server uses the data for estimating the environmental field.

In the discretized area, the representative location of each cell changes based on the variance in that cell. This results in each robot traversing different lengths of paths, which means robots reach their next waypoint at different times. Therefore, the update of the collected data is asynchronous and planning decisions are made using partial information. We bring synchronization between the team of robots by dividing the total time *T* into intervals of *T*_*s*_, where by the end of each interval the robots reach their waypoints. Therefore, this time interval *T*_*s*_ is sufficient for a robot to reach the neighboring cell, even when traversing at the average speed. The addition of this synchronization time step also transforms the decision step from *t* + 1 to *t* + *T*_*s*_ and thus the framework uses this time interval to plan for the paths ahead of the next synchronization event.

### 4.1. Multi-Robot Path Planning With No Constraints

We make use of *single-robot dynamic programming (DP)* along with the spatial decomposition algorithm discussed in Mishra et al. (2018) to explain our multi-robot path planning algorithm. This *single-robot DP* algorithm can be defined as a Markov decision process (MDP). The formulation as a MDP will require states to be defined by the cells, actions as the moves available in each of these cells, the transition probabilities as 1, and the reward function given by *R*(·, ·). Extending the single-robot algorithm to a multi-robot scenario requires two modifications. First, the robots should be coordinated to explore an area in a collaborative manner. This is similar to the problem solved in Singh et al. (2009) using a sequential decision algorithm. This algorithm decides a path for one robot first, which is followed by path allocation to the second robot and then sequentially to the remaining robots. However, we are concerned only with the next waypoint in the case of planning with no constraints. The second necessary modification is to prevent the collision between two robots, which can be achieved by having negative rewards for each robot's current location. For the ease of notation, we denote the representative location of a cell visited by a robot *i* at time *t* as **c**_*t, i*_. As we are interested in planning for one-time ahead, the update rules for multi-robot case can be given as:

(19) $$\begin{array}{r} V\left({\text{c}}_{t,i}\right)=\underset{a\in {\text{A}}_{{\text{c}}_{t,i}}}{\max}\left[R\left({\text{c}}_{t,i},a\right)+\gamma V\left({\text{c}}_{t+T_{s},i}\right)\right]\text{,} \end{array}$$

(20) $$\begin{array}{r} \pi \left({\text{c}}_{t,i}\right)=\underset{a\in {\text{A}}_{{\text{c}}_{t,i}}}{\arg\max}\;V\left({\text{c}}_{t,i}\right)\text{,} \end{array}$$

where

$$R(c_{t,i},a)=\{\begin{array}{ll} \frac{\sigma_{*,c_{t}}}{\left\|c_{t,i}-\;c_{t+T_{s},i}\;\right\|} & \text{ if }c_{t+T_{s},i}\notin x_{t+T_{s},1:H}-\{x_{t+T_{s},i}\}\\ -\epsilon & \text{ if }c_{t+T_{s},i}\in x_{t+T_{s},1:H}-\{x_{t+T_{s},i}\} \end{array}\text{,}$$

**x**_*t*, 1:*H*_ represents the current location of all the robots, **c**_*t* + *T*_*s*_, *i*_ represents the location of the cell that the robot *i* will reach at time *t* + *T*_*s*_, and ϵ is the value of the negative reward. We run one full cycle of policy iteration using DP for robot 1 and obtain the optimal policy given by π^*^(·). Using this policy, we get the future location of robot 1, given by ${\text{c}}_{t+T_{s},1}:=\pi^{*}\left({\text{c}}_{t,i}\right)$ and thus we update **x**_*t* + *T*_*s*_, 1_: = **c**_*t* + *T*_*s*_, 1_. We also update this new location for robot 1 in the location set of all robots **x**_*t* + *T*_*s*_, 1:*H*_. This update of the location in the set of locations **x**_*t* + *T*_*s*_, 1:*H*_ makes sure that robot 2 and the remaining robots do not visit the same cell where robot 1 will be at the next time step. We run such cycles sequentially for all the *H* robots and obtain the next respective waypoints. We name this algorithm the *multi-robot DP*.

### 4.2. Multi-Robot Path Planning With Temporal Constraints

Introducing time constraints to the multi-robot framework explained above is not straightforward. The new waypoints generated using the above framework may not be optimal given the temporal constraints *T* − *t*. Therefore, we need to find a combination of actions for different robots that would reduce the overall variance within the remaining time. Let Φ_*t* + *T*_*s*__ denote this combinatorial set of all actions *A*_**c**_*t* + *T*_*s*_, *i*__ ∀*i* ∈ [1, *H*] at time *t* + *T*_*s*_. We define another combinatorial set φ_*t* + *T*_*s*__, which is a subset of Φ_*t* + *T*_*s*__ representing one action for each robot. From the set Φ_*t* + *T*_*s*__, we remove the states where the next action for two or more robots will result in a collision. Therefore, the optimal combination of action $\varphi_{t+T_{s}}^{*}$ at time *t* + *T*_*s*_ can be given by:

(21) $$\begin{array}{r} \underset{\varphi_{t+T_{s}}^{\prime }\in \Phi_{t+T_{s}}}{\arg\max}\;U\left(\varphi_{t+T_{s}}^{\prime }\right)+\eta \vartheta_{T-\left(t+T_{s}\right)}\left(\varphi_{t+T_{s}}^{\prime }\right)\text{,} \end{array}$$

where $U\left(\varphi_{t+T_{s}}^{\prime }\right)$ is a function that gives the sum of variances of cells that will be visited due to the combination of actions in $\varphi_{t+T_{s}}^{\prime }$, η is a discounting factor, and $\vartheta_{T-\left(t+T_{s}\right)}\left(\varphi_{t+T_{s}}^{\prime }\right)$ represents the potential of reducing variance within the remaining time *T* − (*t* + *T*_*s*_) by taking the combination of actions given by the set $\varphi_{t+T_{s}}^{\prime }$.

Interestingly, calculating the variance has no direct dependency on the target values **y**_*t*:*T*_ as shown in (5). This suggests that once the kernel function is learned using the collected data, we can estimate the change in variance over the field. We use this characteristic to get an estimate of $\vartheta_{T-\left(t+T_{s}\right)}\left(\varphi_{t+T_{s}}^{\prime }\right)$. The variance after taking a path can be estimated using (5) and the remaining overall variance in the field will give the estimate for $\vartheta_{T-\left(t+T_{s}\right)}\left(\varphi_{t+T_{s}}^{\prime }\right)$. However, obtaining just this value will not solve our problem. We need to coordinate a team of robots and select the best available option given the remaining time *T* − (*t* + *T*_*s*_). We still need to search through the set Φ_*t* + *T*_*S*__ to select a set of actions at time *t* + *T*_*s*_.

**Algorithm 1:**
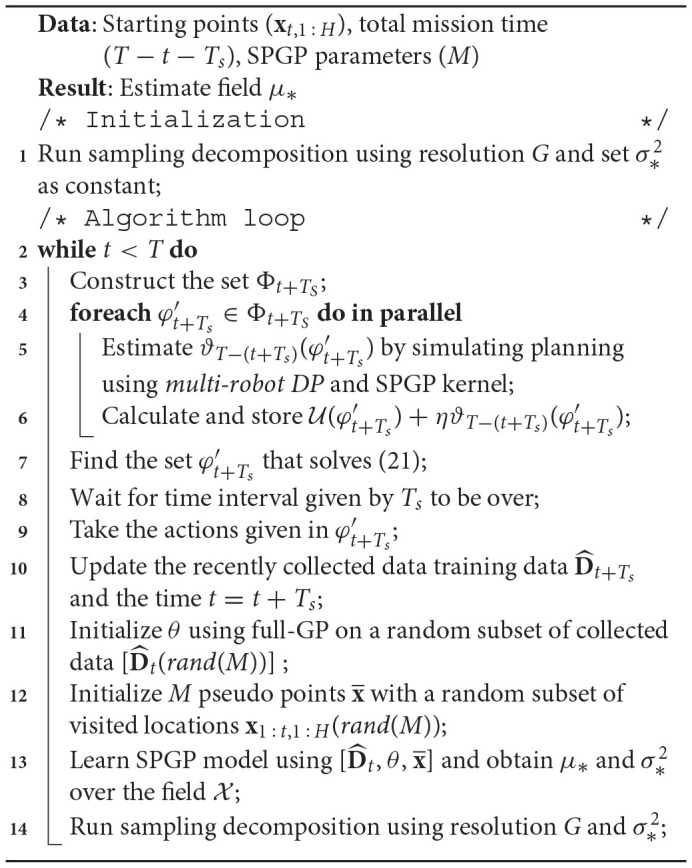
*m-AdaPP* - multi-robot adaptive path planning

**Figure 2:**
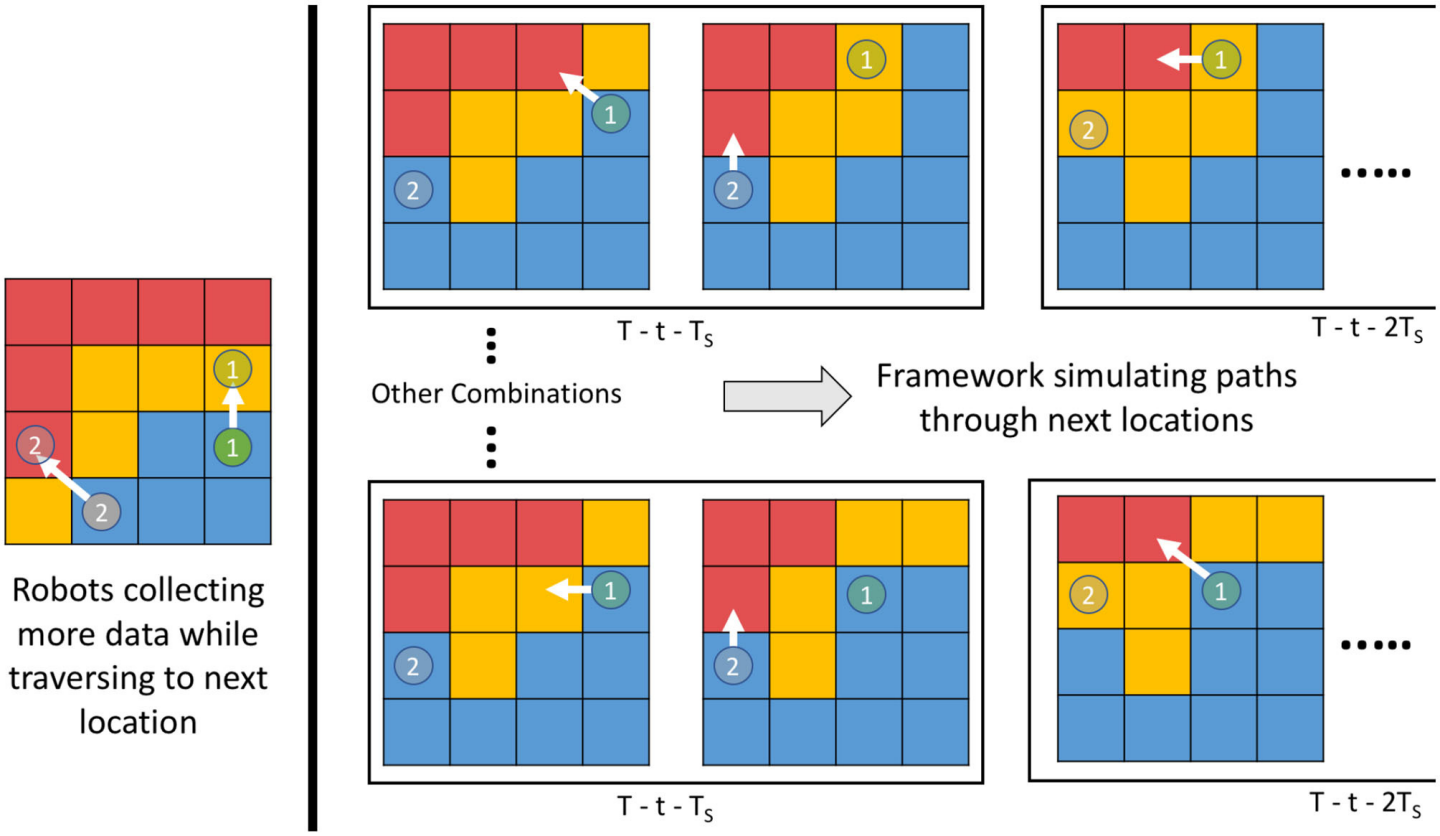
A concept diagram explaining the sequential planning in our multi-robot framework *m-AdaPP*. The left-most grid shows the robots traversing a path to the next waypoint. In parallel, the framework is planning for the next of actions assuming the robots have already reached the location. The framework simulates paths and updates the variance for the remaining time and select the actions that minimize the overall variance.

**Figure 3:**
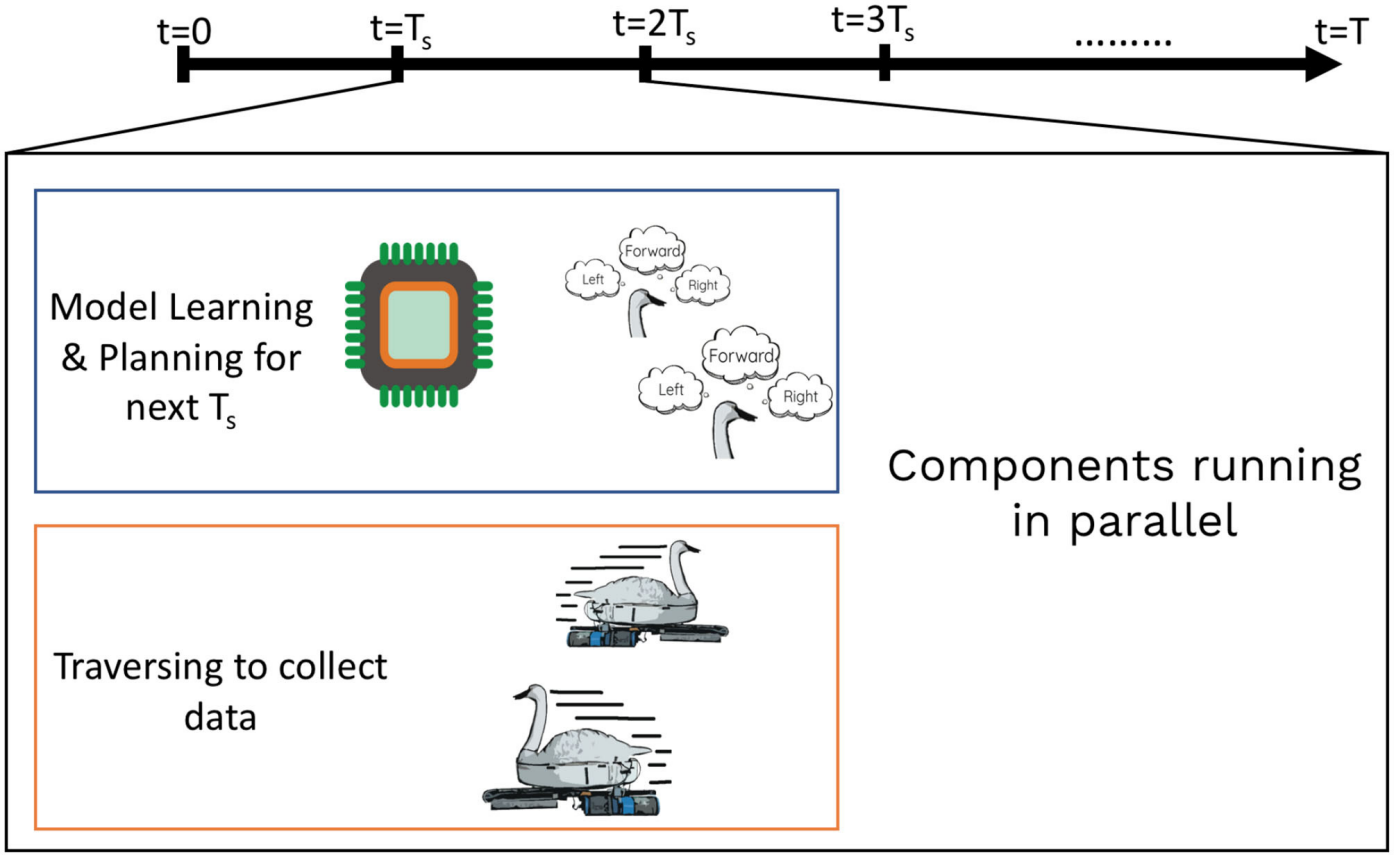
An illustration showing execution of one step of our framework. The GP model learning and planning for the next waypoint occurs in parallel while the robot is collecting data from the field. Such an approach can be used for efficient use of mission time.

We solve the problem of coordination between the robots by using *multi-robot DP* at each simulated planning iteration and provide a combination of actions. We do this in two steps. First, we run one full iteration of *multi-robot DP* and obtain a set of actions $\varphi_{t+T_{s}}^{\prime }$. Second, we reduce the total time by *T*_*s*_ and update the variance of the cells based on the paths the robots will take due to the actions given by $\varphi_{t+T_{s}}^{\prime }$. We re-run the *multi-robot DP* algorithm to find the next set of actions $\varphi_{t+2T_{s}}^{\prime }$ using the updated variance. We iterate over these two steps until the mission time is over *t* = *T*. Using this approach, we get an estimate of ϑ_*T* − (*t* + *T*_*s*_)_(·) and thus we can evaluate the value of the combination $\varphi_{t+T_{s}}^{\prime }$ given by (21). Similarly, we can use this to find the values for all the combinations given by the set Φ_*t* + *T*_*s*__. Once we have the values for all the actions, we can use (21) to find the set of actions for the robots for time *t* + *T*_*s*_. An example of these steps is illustrated as a diagram in Figure 2. All these steps are repeated whenever the training dataset ${\hat{\text{D}}}_{t}$ is updated, which will be at a regular interval of *T*_*s*_ and thus bring the adaptive nature to the *m-AdaPP* framework. Our overall framework is presented in Algorithm 1 and a graphical illustration of it is shown in Figure 3.

There are two important points to note about our framework. First, the decisions are made sequentially but it does not mean the robots also move sequentially. Once a decision is made, all the robots move to their next location simultaneously within time *T*_*s*_. Second, the calculation of ϑ_*T* − (*t* + *T*_*s*_)_(·) for one set of action in Φ_*t* + *T*_*s*__ is independent of the other set of action. This provides an opportunity to estimate the value for ϑ_*T* − (*t* + *T*_*s*_)_(·) for all the sets of actions in parallel. This helps in reducing the overall computation time of our framework.

### 4.3. Field Prediction Using SPGP

We make use of the same kernel function used in single-robot frameworks. It is defined by *K*(·, ·):

(22) $$\begin{array}{r} K\left({\text{x}}_{n},{\text{x}}_{n^{\prime }}\right)=\alpha \exp\left(\frac{1}{2}\overset{2}{\underset{l=1}{\sum }}b_{l}{\left(x_{n,l}-x_{n^{\prime },l}\right)}^{2}\right)\text{,} \end{array}$$

where α, *b*_1_, and *b*_2_ are the parameters of the kernel function, **x**_*n*_ and ${\text{x}}_{n^{\prime }}$ represent two different locations, and *x*_*n, l*_ represents the value for the *l* dimension of **x**_*n*_. After adding the Gaussian noise model, the hyperparameters of the sparse GP are given by $\theta =\left\{\alpha ,b_{1},b_{2},\sigma^{2}\right\}$ and pseudo inputs $\bar{\text{x}}$. Following the suggestions given in Snelson and Ghahramani (2006), we initialize the pseudo points with random spatial locations from the collected data and initialize the kernel hyperparameters by learning a full-GP model with the same kernel function but using only a small subset of the dataset.

## 5. Experimental Results

We performed two sets of experiments to test the performance of our framework. We first examined the coordination within the team of robots and later we did experiments in Singapore waters to compare the fields estimated by our framework and lawn mower patterns. Finally, we examined the biological relevance of the fields estimated using our framework.

### 5.1. Simulations to Test the Coordination Efficiency

**Figure 4:**
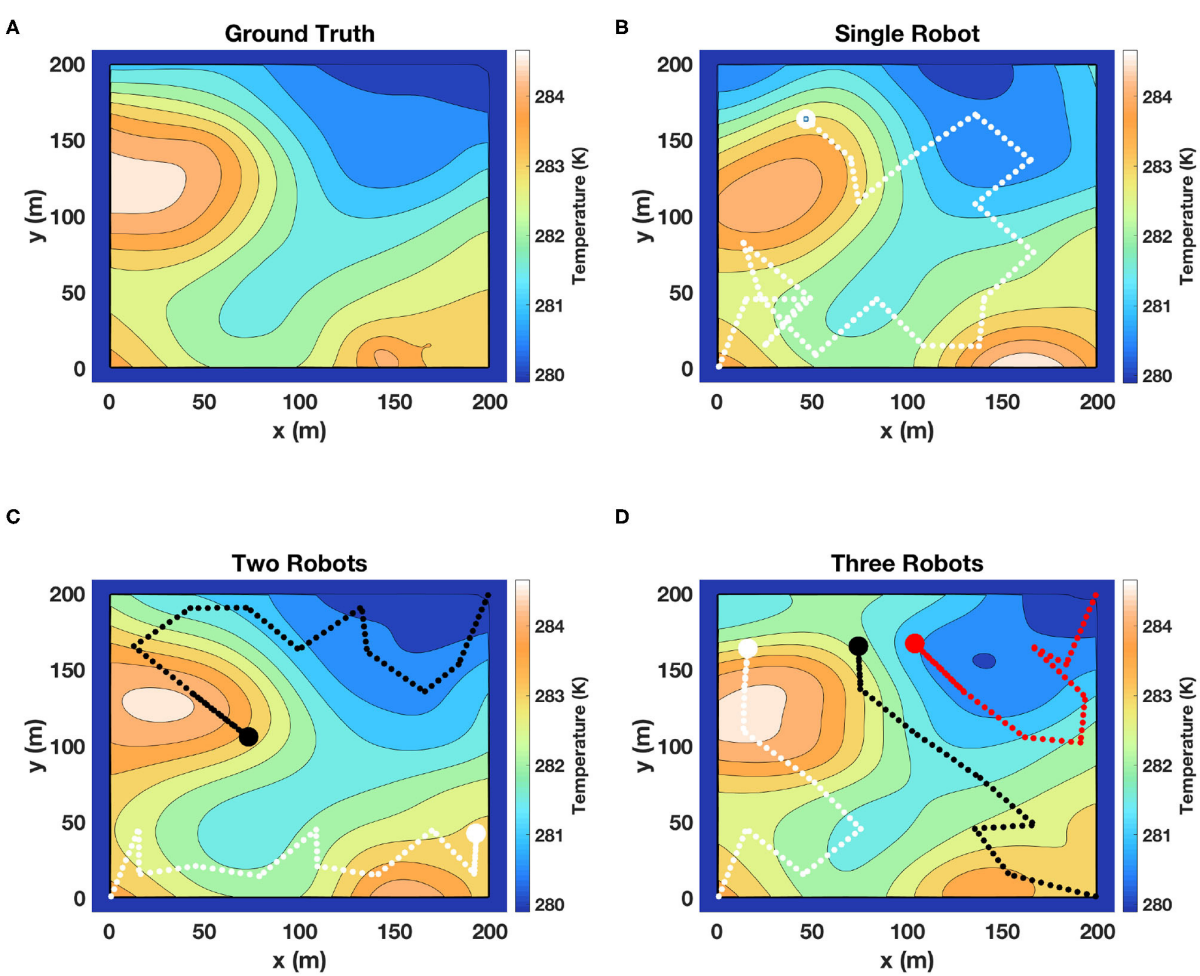
Simulation results of *m-AdaPP* for estimating a temperature field. **(A)** Represents the ground truth and **(B)** represents the field estimated using one robot. Similarly, **(C,D)** represent the field estimated using two robots and a team of three robots, respectively. The mission time for **(B)** is *T* = 2, 400 s, **(C)** is *T* = 1, 200 s, and **(D)** is *T* = 800 s. It can be observed that the hot and cold regions estimated using different teams of robots are correct. This shows that our framework efficiently coordinates the team of robots and makes efficient use of mission time to collect good representative data.

**Figure 5:**
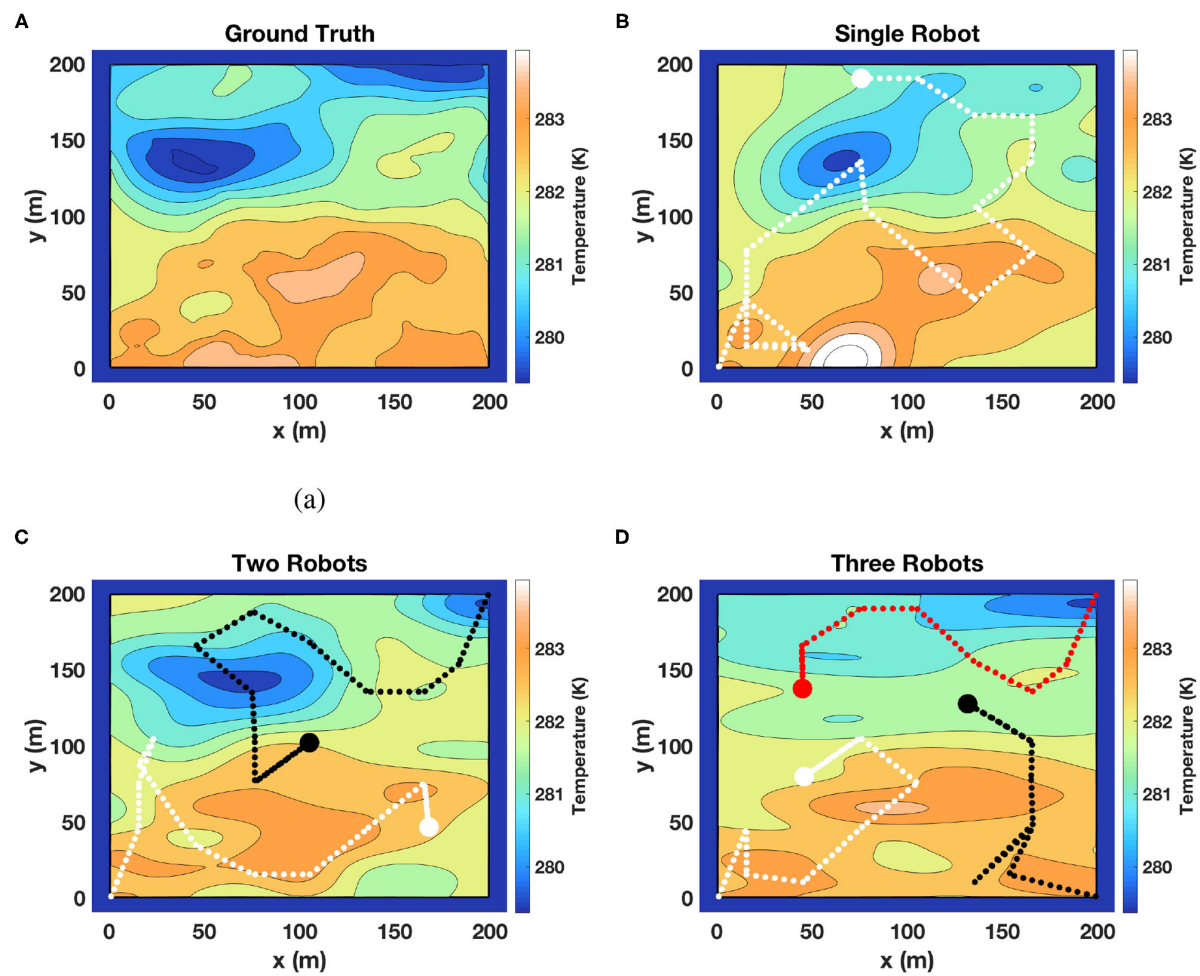
Another set of results of our framework on estimating a temperature field from the SST dataset. **(A)** Represents the ground truth and the remaining figures **(B–D)** represent the field estimated using one, two, and three robots, respectively. Similar to the previous figure, the fields estimated using different teams of robots are comparable. These results are another example showing that our framework coordinated the team of robots well.

In our previous work (Mishra et al., 2018; Mishra, 2019), we have shown via simulations that our single robot adaptive algorithm performs better as compared to lawn mower and other commonly used search techniques. The objective of these simulations was primarily to establish that our framework is capable of coordinating a team of robots and provide a good estimate of the field. We used field data of sea surface temperature (SST) provided by the Jet Propulsion Laboratory (JPL MUR MEaSUREs Project, 2010). We extracted data for two regions of 200 × 200 km^2^ each, and mapped each to a field with an area of 200 × 200 m^2^. This scaling was done to retain essential features of a scalar temperature field, but also to include an area which can be explored within a practical value of mission time *T*. The main feature of this field is its scalar nature and not that it represents the sea surface temperature. It can be easily compared to the fields of vegetation spread, air quality, or ash plumes. We denote the two scaled temperature fields shown in Figure 4A as Field 1 and Figure 5A as Field 2 for the following discussions.

The maximum speed of the robots used in environmental monitoring is generally low. This is to make sure that the robots do not cause substantial disturbance to the environment they is sensing. For example, the maximum speed of our water-quality sensing robot, NUSwan (Koay et al., 2017), is 1 m/s. However, the average speed of NUSwan with external disturbances such as strong winds or waves is about 0.3 m/s. We use this speed to define the value of *Tcleveref*_*s*_. Following the grid size *G* = 30 m, the average time required for traveling from one cell to another cell will be at least 100 s. Therefore, we set the value of *T*_*s*_ as 120 s giving the vehicle sufficient time to reach the next cell.

We learned the SPGP model with *M* = 50 pseudo data points. Similar to the single robot framework, we initialized the pseudo points with *M* random points of the total dataset and ran a full GP regression to initialize the hyperparameters of our kernel function. The simulation experiments were implemented in MATLAB. For SPGP, we took the MATLAB code provided by the authors (Snelson and Ghahramani, 2006) and modified it for spatial regression application. The simulations were run on a hexa-core Intel i7 processor with 32 GB of RAM.

We simulated teams consisting of a maximum of three robots. We examined the coordination within the team of robots by providing less mission time for the teams with a higher number of robots. This means that the team with two robots has less time compared to a single robot. If the framework is able to coordinate the team of two robots well, the performance of these two simulations should be comparable. For our simulation setup, we set the mission time *T* as 2, 400 s for a single robot, 1, 200 s for a team of two robots, and 800 s for a team of three robots.

Note that the mission time *T* for a single robot here is 2, 400 s, which is much higher than the mission times set in our previous work (Mishra et al., 2018). This difference is due to the assumed vehicle speed, and a relationship can be seen in terms of distance traveled: a vehicle with speed 0.3 m/s travels around 700 m in 2, 400 s. Whereas, the same vehicle with an increase of 1 m/s in speed travels the same distance in 700 s. Therefore, our limit on mission time in the current setup is not substantially different from the setup in Mishra et al. (2018). Moreover, our average computation time for the team of three robots after parallelization was about 23 s, which is much less than *T*_*s*_ and thus satisfies the constraint on τ given by (16).

**Figure 6:**
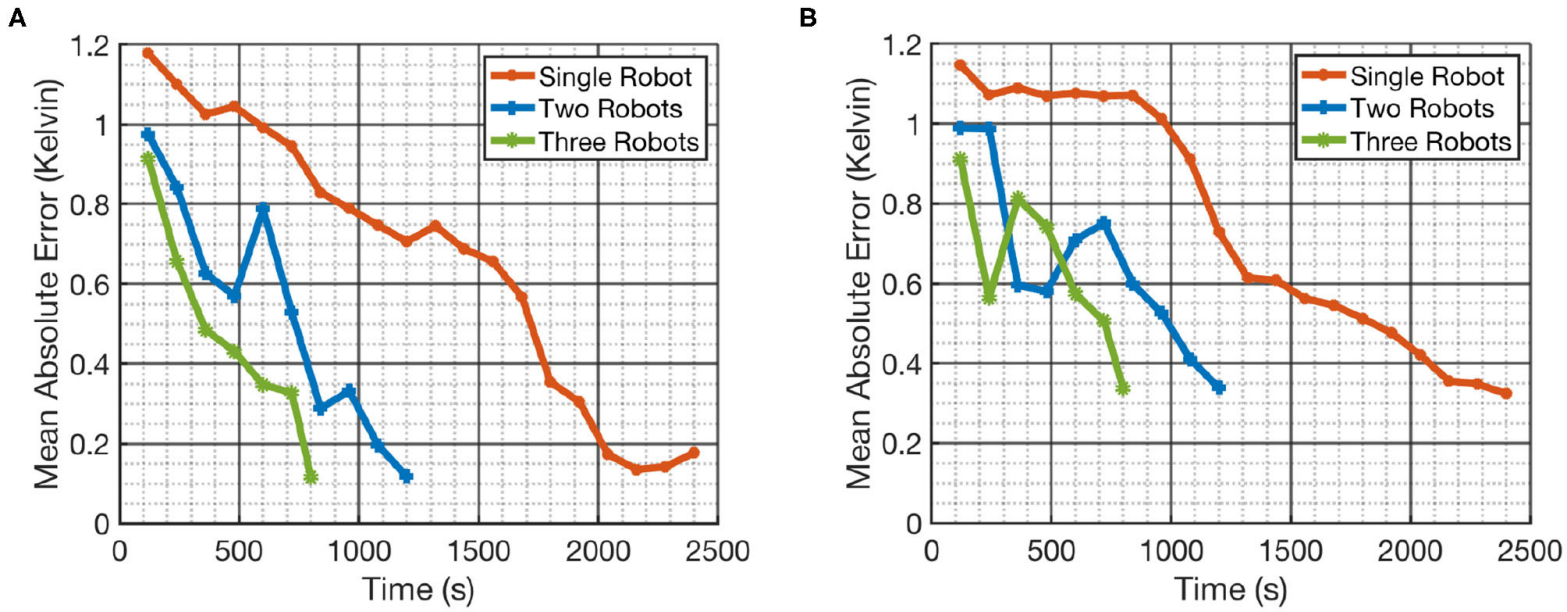
Mean absolute error (MAE) in estimating the temperature field using different teams of robots. **(A)** Shows the error in estimating the field given by Field 1 and **(B)** shows the error in estimating Field 2. The similar MAE values for different teams of robots with different mission time *T* provide more objective evidence that our framework is capable of coordinating the teams well.

The results of the fields estimated using *m-AdaPP* are shown for one simulation run in Figures 4, 5. It is clear from the figures that the estimated hot and cold regions in our framework are correct and the overall estimated fields are similar for teams with different numbers of robots. We also calculated the mean absolute error (MAE) over all the locations in the entire field and used it as a measure of performance in estimating the fields. We use this metric to examine the coordination efficiency of our framework. The MAE results for one simulation run are presented in Figure 6.

**Figure 7:**
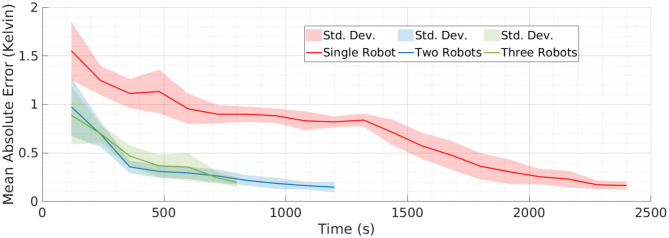
The mean absolute error over 10 runs for different teams of robots. The result shows the error in estimating the field given by Field 1. The consistent performance of our multi-robot framework over multiple runs provides the evidence that our framework is robust.

It can be observed from Figure 6 that our framework's performance is similar for different teams of robots. The mission time for each team of robots is proportional to the number of robots in each team. This means that the amount of data collected by a single robot in *T* = 2, 400 s will be similar to the amount of data collected by a team of two robots in *T* = 1, 200 s. A similar performance between these two setups will show that our framework is able to efficiently coordinate the team of robots. Therefore, the similar MAE values in Figure 6 for different teams of robots and for different fields is a good indication that our framework is capable of coordinating the team efficiently. It can be also observed from Figure 6 that the performance of multi-robot teams is less monotonic. This could be due to the random initialization of SPGP and thus we also repeated the simulations over 10 runs for each team of robots and recorded the MAE. The main difference between these 10 runs was the random initialization of the SPGP model and the corresponding planning using this SPGP model. These results are presented in Figure 7, and it can be observed that our framework shows a consistent monotonic performance over multiple runs. The results in Figure 7 give an overview of the performance over 10 runs whereas Figures 4–6 are results from a randomly selected instance.

### 5.2. Performance Comparison With Greedy IPP in Simulations

Our framework searches for a combination of actions for the team of robots that satisfies (21). This equation includes both the short term goals, denoted by U(·), and the long term goals, denoted by ϑ_*T* − (*t* + *T*_*s*_)_(·). Interestingly, removing the term ϑ_*T* − (*t* + *T*_*s*_)_(·) from (21) will shift the framework's focus to the sum of variance of neighboring cells and thus convert our framework into a greedy IPP. Moreover, removing this term will also relax the dependence on future moves and thus simulate a myopic planning approach. The time bounds will only be present to stop the simulation and not constrain the framework's planning or model learning.

The key difference between the greedy IPP and our framework is the selection of actions at any given time *t*. Both the frameworks use the same sparse GP method and the actions are selected in a centralized manner. The performance of this greedy framework can be thus used as a benchmark and effectively compare two different IPP approaches, myopic and non-myopic.

**Figure 8:**
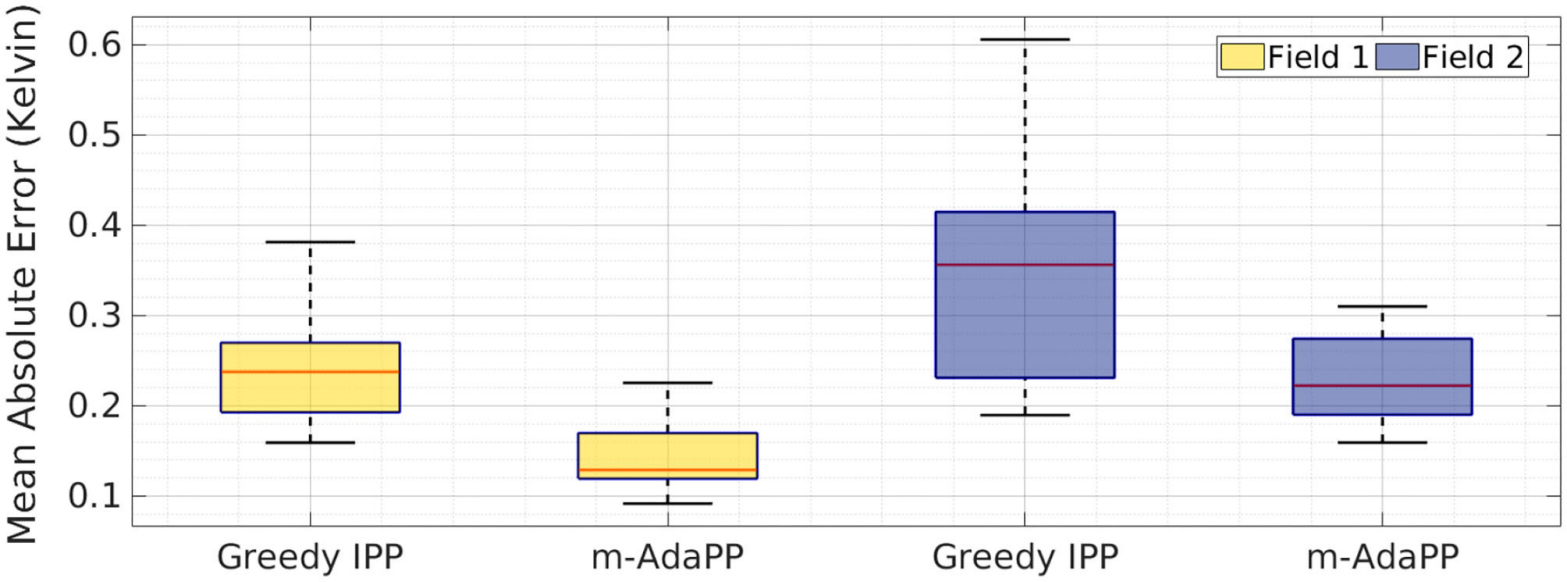
Mean absolute error over 10 runs shown as a boxplot for a team of two robots with greedy IPP and *m-AdaPP*. The error calculated in estimating both the fields, Field 1 and Field 2. The greedy IPP is a multi-robot IPP that aims to reduce the maximum sum of variance in the neighboring cells, and hence simulates greedy planning. The error values show that *m-AdaPP* performs better compared to the greedy IPP.

We simulated the greedy IPP using the simulation setup explained in the previous section. The greedy IPP and *m-AdaPP* are both given the same amount of time for a team of two robots and we simulated 10 runs for both the fields. We calculated the MAE values for all the runs and the end results are shown in Figure 8. It can be clearly observed that *AdaPP* performs better when compared to a greedy IPP. These results are encouraging as it shows that our non-myopic planning approach performs well and efficiently coordinates the team of robots within the given time.

### 5.3. Simulations to Compare Performance With a Distributed Implementation of *m-AdaPP*

Our framework makes use of centralized planning for coordinating the team of robots and this centralized planning can be distributed for the team of robots using different approaches. One of these approaches can be splitting the area into proportional areas to the number of robots and perform planning for each robot in its respective area. It is important to note that in this approach only the planning will be performed separately for each robot and the model learning will still be centralized. The use of such a distributed planning approach will decouple the next action selection for each robot, however, it will also put restrictions on the coordination of robots as the framework can only use one robot in the designated area.

**Figure 9:**
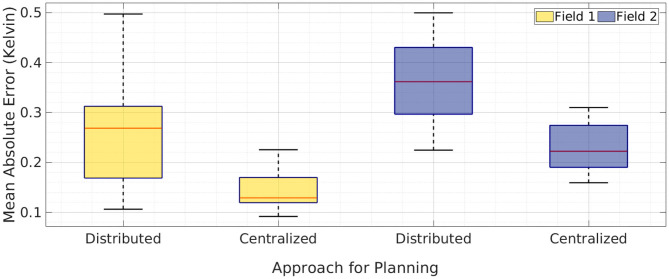
A boxplot of mean absolute error over 10 runs for a team of two robots with distributed planning and centralized planning of *m-AdaPP*. The result shows the error in estimating both the fields, Field 1 and Field 2. The tiled version of AdaPP for a team of two robots is essentially separating the field into two equal halves and using *m-AdaPP* for planning individually. It is important to note that model learning in distributed planning and centralized planning of *m-AdaPP* is the same and only the approach for planning is different. The error values show that centralized planning performs better when compared to the tiled version.

We simulated this distributed planning approach to compare its performance with the suggested centralized framework. We used the same setup as described in section 5.1 for a team of robots. Field 1 and Field 2 were both split into two equal left and right halves for simulating the distributed planning approach. We simulated 10 runs for both the fields and used both the versions of our planning approach. The MAEs for these simulations are shown as boxplots in Figure 9. It can be clearly observed from the boxplots that median errors of centralized planning are about half the median errors of distributed planning. This result shows that our centralized planning performs better when compared to the distributed planning.

### 5.4. Field Experiments for Performance Comparison With Lawn Mower Paths

**Figure 10:**
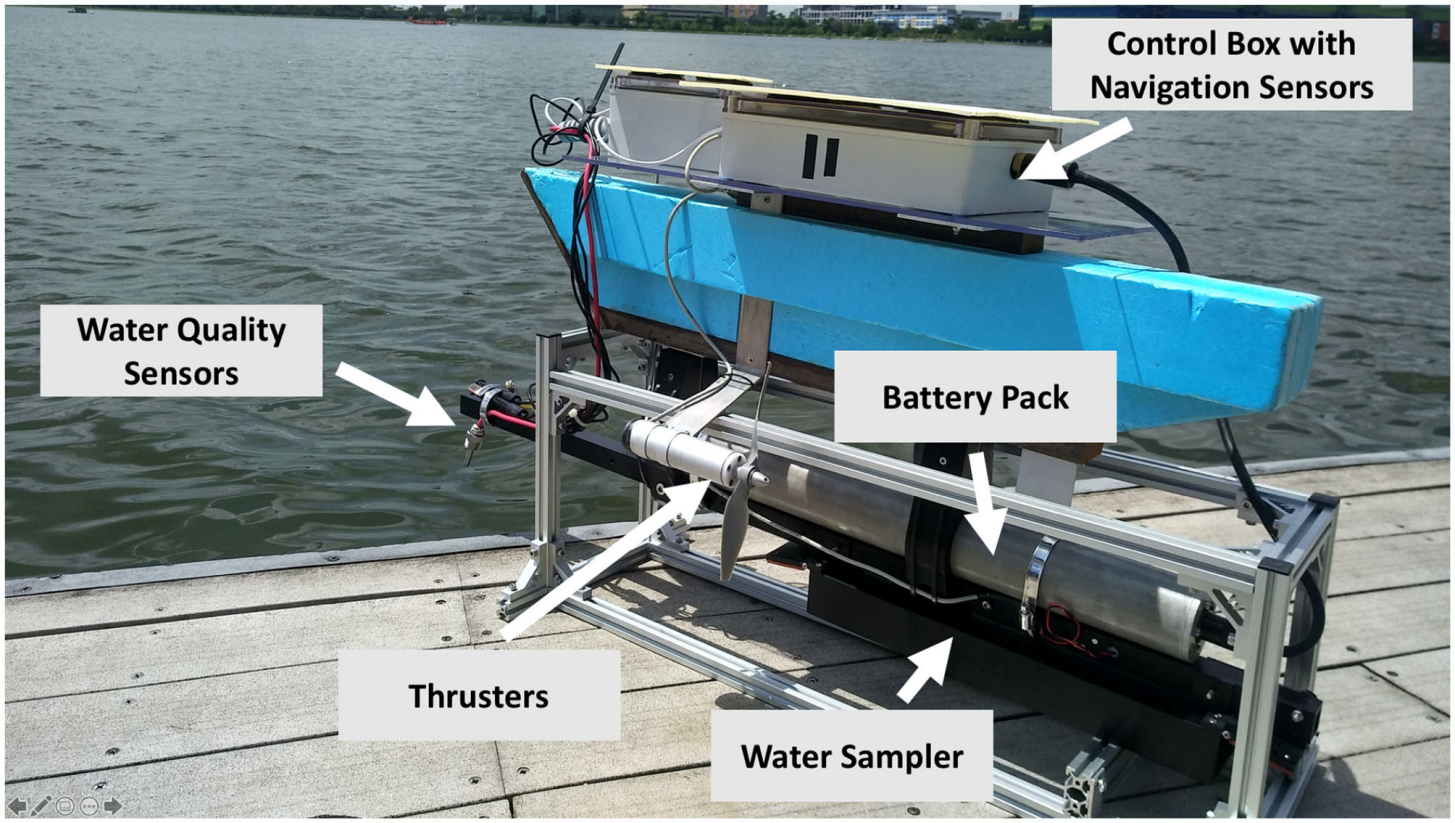
One of the robots we used in our field experiments. It is a variant of NUSwan (Koay et al., 2017). This figure shows various components present in our robot. Our robots are capable of navigating autonomously once a waypoint is given. It is equipped with general water-quality sensors and provides real-time updates of the physical and chemical parameters of water. Moreover, our robots use a middleware which enables them to receive waypoints from remote servers and provide the mission relevant information back to the server for future planning.

We tested the performance of our framework against conventional approaches such as estimating fields using lawn mower paths via field experiments. We developed two variants of the NUSwan (Koay et al., 2017) robot as shown in Figure 10. These robots were equipped with general water-quality sensors such as DO, conductivity, pH, and oxidation-reduction potential. Moreover, these robots used on-board navigation sensors to guide the robot to the locations given by the framework. Our framework *m-AdaPP* was hosted on a cloud server, which can be accessed by our robots using a mobile network. This cloud server was a compute instance provided by Amazon Web Service with the capability to run 16 threads in parallel. This capability is crucial for our framework as it significantly reduces the computation time for making planning decisions. We optimized our framework to run smoothly on this compute instance. Both the robots posted the data to this server every 5 s.

For consistency, the mission time for the team of two robots for our field experiments is the same as the mission time we used for two robots in our simulations, which is *T* = 1, 200 s and *T*_*s*_ = 120 s. In general, lawn mower paths are defined by the number of legs, where each leg is a straight path parallel to one of the axes of the survey area. If the speed of the vehicle is constant, lawn mowers can be defined in terms of time but speed of the vehicle in the field can vary due to external disturbances. Therefore, the lawn mowers are defined in terms of lengths rather than time.

Imposing the temporal constraints directly on the lawn mower paths can result in abruptly stopping the lawn mower pattern. Therefore, we assume an average speed of the robots and use this average speed to calculate the total length of the lawn mower for the mission time *T* = 1, 200 s. We set this average speed as 0.5 m/s. Note that this average speed is higher than the average speed mentioned earlier. This difference is to factor in the fact that the vehicle mostly moves in a straight line and thus inertia of the vehicle helps in maintaining a higher speed. Using the average speed of 0.5 m/s and a mission time of *T* = 1, 200 s, we set the length of the lawn mower as 600 m.

**Figure 11:**
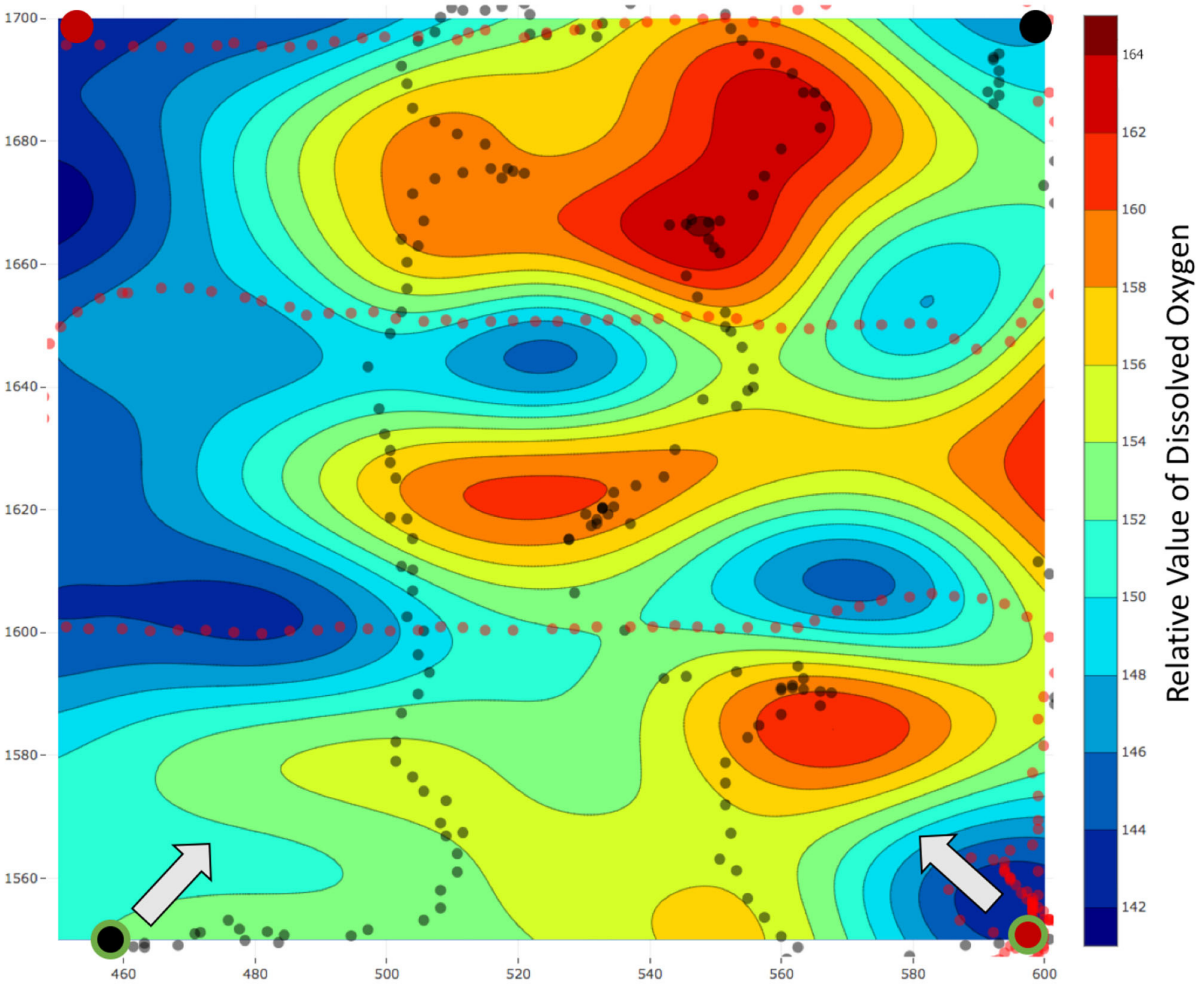
Field estimated using lawn mower patterns with a team of two robots. The estimated field is for relative dissolved oxygen for an area of 150 × 150 m^2^ in a local reservoir. The black and red circles with a large radius and no outline represent the starting locations of the robots. Similarly, the black and red dots represent the locations of the data collected. Finally, the black and red circles with a large radius and a green outline reflect the end location of each robot and the arrow represents the direction toward the starting location. The total mission time for this experiment was *T* = 1, 236 s.

**Figure 12:**
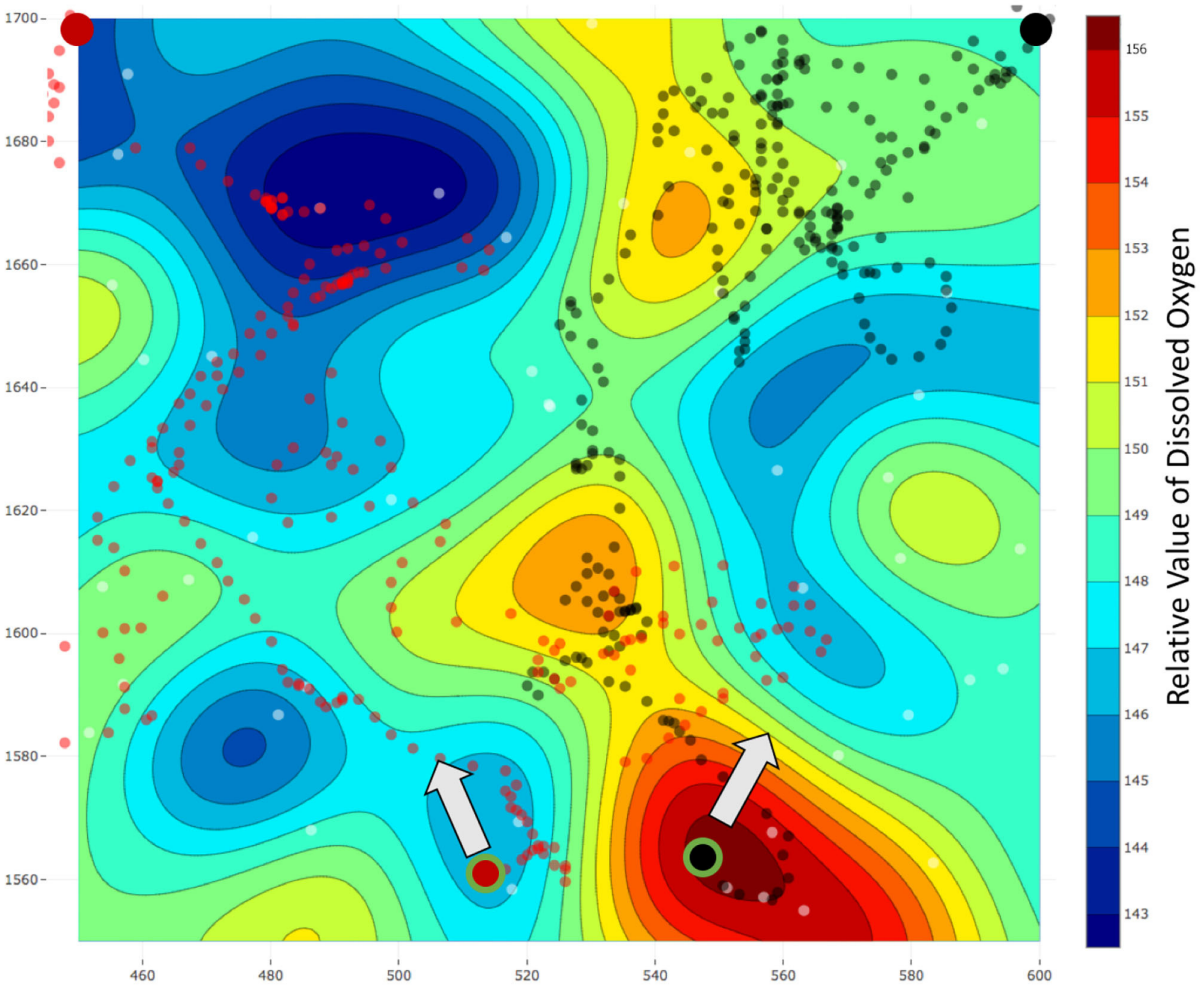
Field estimated using a team of two robots and our framework *m-AdaPP*. The estimated field is for relative dissolved oxygen for an area of 150 × 150 m^2^ in a local reservoir. The black and red circles with a large radius and no outline represent the starting locations of the robots. Similarly, the black and red dots represent the locations of the data collected. Finally, the black and red circles with a large radius and a green outline reflect the end location of each robot and the arrow represents the direction toward the starting location. The total mission time for this experiment was *T* = 1, 200 s with *T*_*s*_ = 120 s. It is interesting to observe that data collected using this team of robots were dense in a few regions, whereas, sparse for the remaining regions. However, our framework still performs better as compared to the lawn mower pattern and this is a field-validated result that collecting representative data (adaptive framework) can perform better when compared to collecting data with repetitive information (lawn mowers).

We selected a survey field of area 150 × 150 m^2^ in a local reservoir and used our robots to estimate the field of DO over this area. The estimated fields using the lawn mower patterns and our frameworks are present in Figures 11, 12, respectively. The mission time for the lawn mower paths was 1, 236 s and thus our assumption of a higher average speed was correct. Additionally, the distance traveled by the robots while using our framework is less when compared to lawn mowers, generally within a 5% range. The distance traveled is less mainly due to momentary stops during synchronizations between the team of robots and the server. The black and red circles with a large radius and no outline represent the starting locations of the robots in Figures 11, 12, whereas, the circles with a green outline represent the end location of the robots.

We collected a test dataset to measure the performance of our framework and the lawn mower paths. This test dataset was collected while robots were traveling back to the starting location after finishing the mission. This dataset contained both the locations as well as the ground truth data for the respective locations. We obtained the estimated DO value for these locations using the learned models and calculated the errors using the collected ground truth data. Additionally, we calculated the mean and standard deviation for each of the collected test datasets. These statistical values can be used to approximate the similarity between the two datasets. The results for both are presented in Table 2. It can be observed that both the test dataset had similar characteristics and thus the errors of the two methods can be compared. The calculated errors for our framework are significantly lower compared to the errors for the lawn mower paths. These field experiments demonstrate that our framework is able to provide a better estimate of the environmental field.

**Table 2:** The root mean square error (RMSE), mean absolute error (MAE), and statistics for the test dataset used for each approach.

**Estimated using**	**Estimation error**		**Test data**	
	**RMSE**	**MAE**	**Mean**	**Std. deviation**
Lawn mower patterns	6.6	4.8	148.4	5.2
*m-AdaPP*	3.9	2.8	149.1	4.5

*These values represent the error in the estimation of the dissolved oxygen field. These errors were calculated using the test data collected by the team of robots while returning to the starting location and the mean and standard deviation for each of the test dataset is available in the last two columns. Our framework gives about a 50% improvement in performance as compared to the fields estimated using lawn mowers*.

### 5.5. Using Estimated Fields for Scientific Experiments

**Figure 13:**
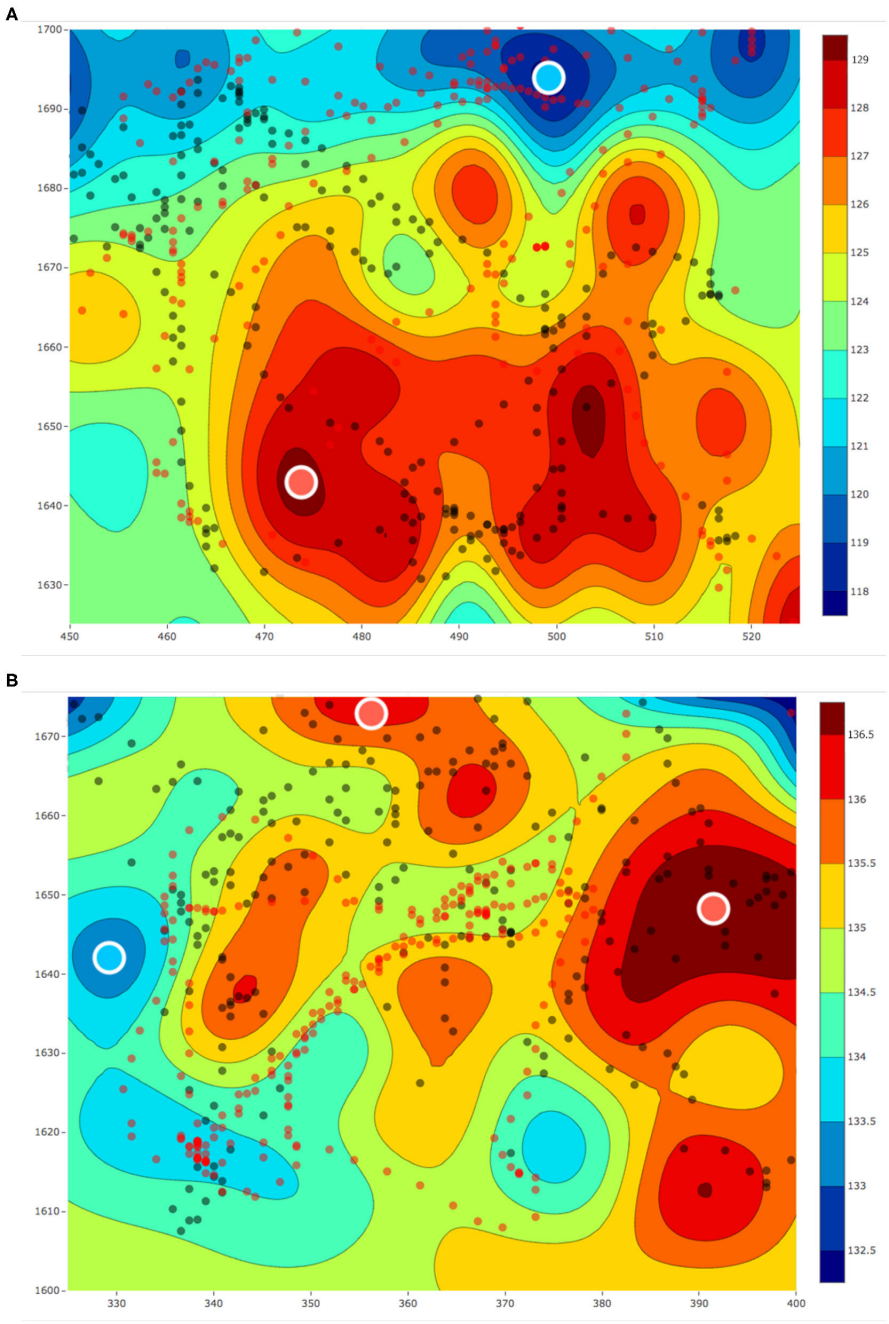
**(A)** The estimated fields of relative values of dissolved oxygen for a 75 × 75 m^2^ area in Pandan Reservoir on March 4, 2019 at **(A)** 01 : 05 p.m. and **(B)** 02 : 25 p.m. The red and black dots, respectively, represent the paths of the two robots. The red and blue circles with the white outline represent the samples collected from the hot and cold regions, respectively.

**Figure 14:**
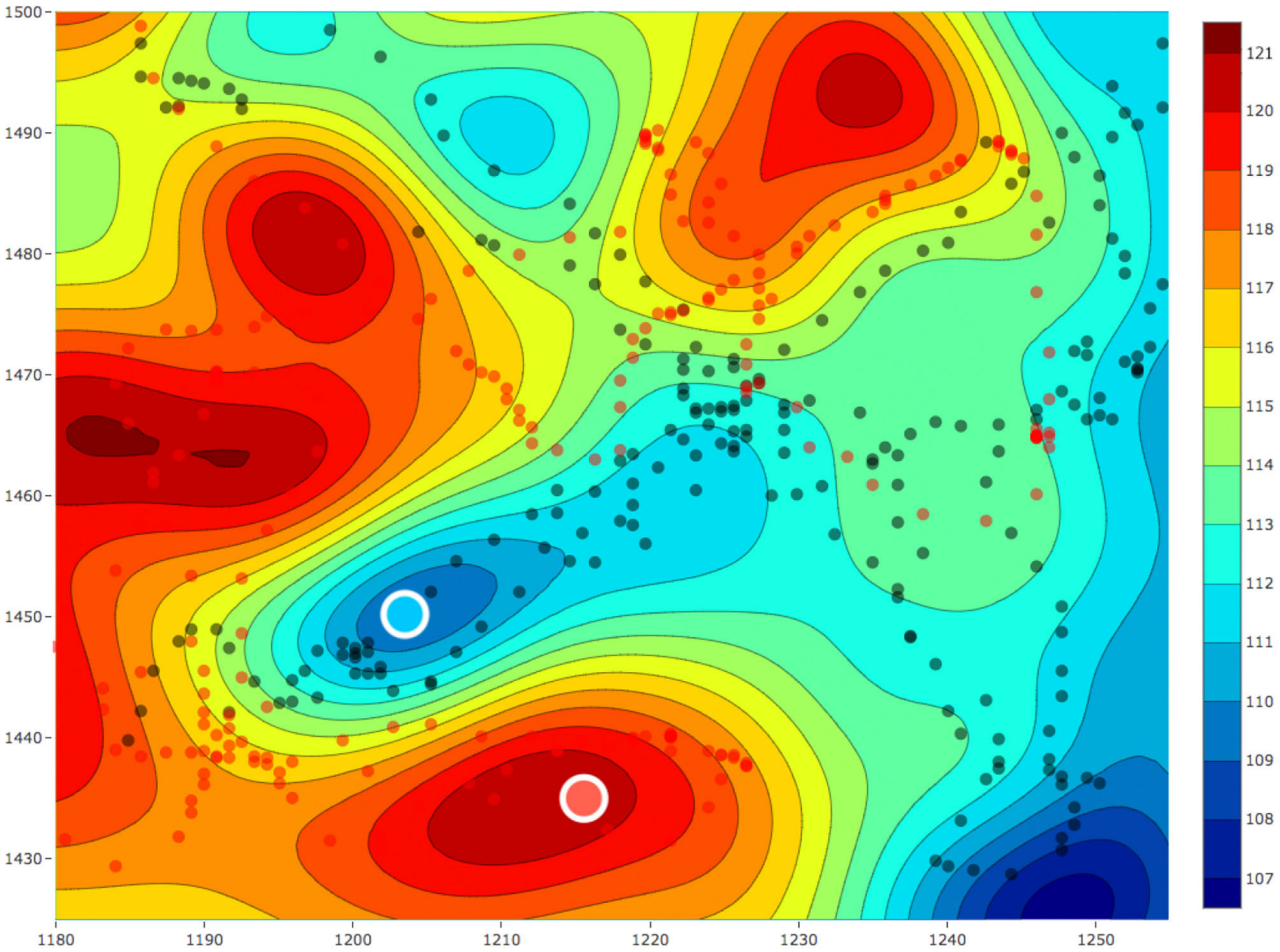
The estimated field for relative value of dissolved oxygen for a 75 × 75 m^2^ area in Pandan Reservoir on February 28, 2019. The red and black dots respectively represent the paths of the two robots. The red and blue circles with the white outline represent the samples collected from the hot and cold regions, respectively.

The aim of these experiments was to use the estimated fields for selecting locations to collect water samples from different concentrations of a water-quality parameter and use these water samples to understand the micro-level interactions. For the sample collection process, we performed three field estimation tasks using our framework. Two out of these three estimation tasks were on the same day with a temporal difference of 1 h. Each of these estimation tasks were given a mission time of 20 min. The following was the overall schedule of our experiments: 10:30 am on February 28, 2019, 01:05 p.m. on March 4, 2019, and 02:25 p.m. on March 4, 2019. These estimated fields are shown in Figures 13, 14, where all the values of dissolved oxygen (DO) are a relative measure of DO instead of the true values.

After each field was estimated, we manually selected the locations to sample and used the robot's automated sampler to collect 1 L of water. These sampling locations are shown as red and blue circles with white outlines in Figures 13, 14. In total, we collected three samples from the regions with low DO values (cold regions) and four samples from the regions with high DO values (hot regions). These samples were then sent for lab analysis such as sample filtering, DNA sequencing, and assembling the DNA to identify different microorganisms. We used the PHRED quality score (Ewing and Green, 1998; Ewing et al., 1998) for our samples. This score is a value between 2 and 40 and it is used to check the quality of the samples before performing any further analysis. This value will be low if the amount of information such as the total DNA present in the samples is not enough to construct and identify the microorganisms. Similarly, this value will be high if the amount of information present in the collected samples is enough for further analysis such as identifying microorganisms. The PHRED quality score can vary due to many different reasons such as sampling location or the filtering process, and thus having an objective score makes it easier to evaluate the samples collected. The mean scores after denoising was approximately 30.

**Figure 15:**
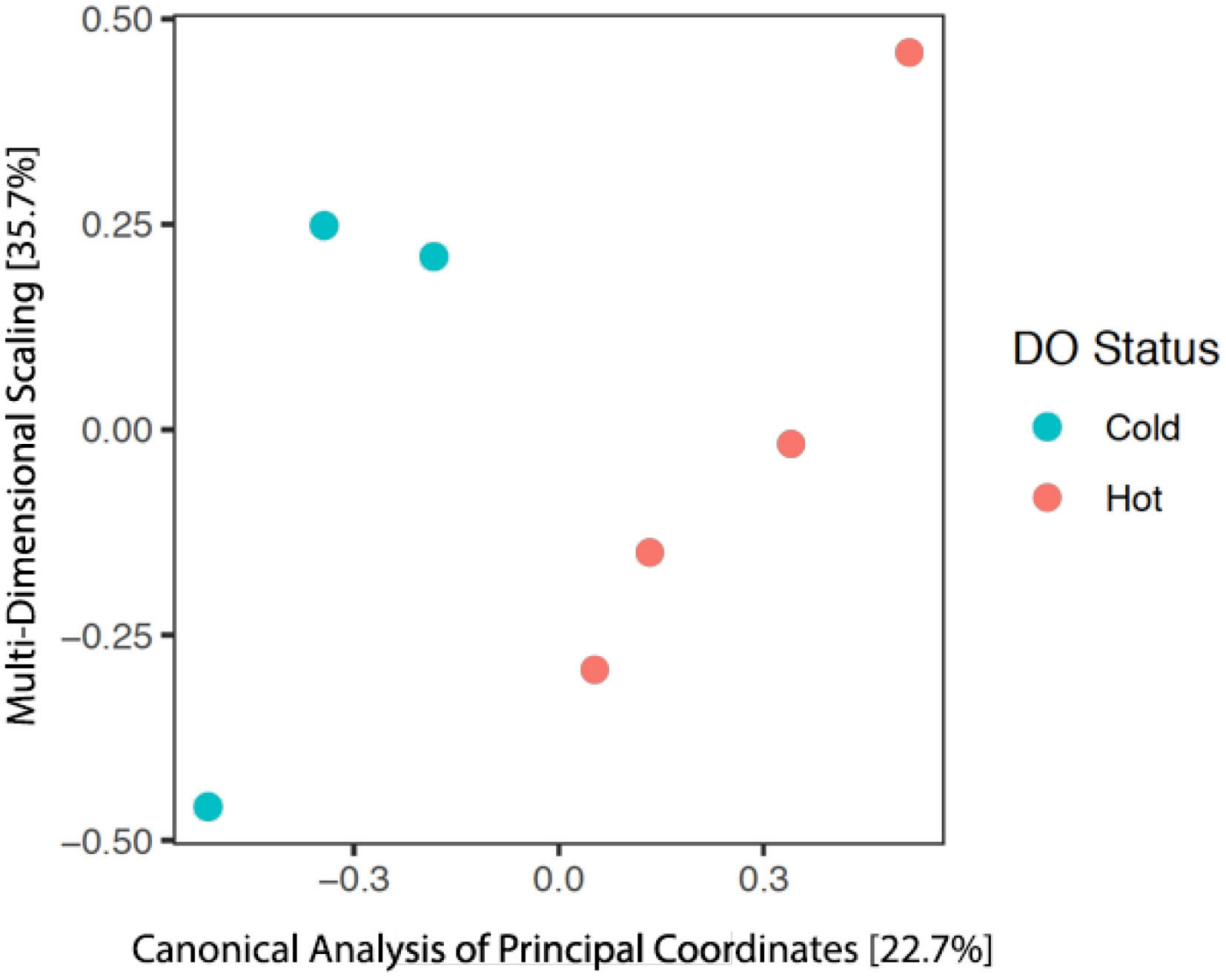
The results from the analysis of principal coordinates and ordination for the microbial communities within each of the seven samples. The red dots represent the samples collected from the hot regions, whereas, the cyan dots represent the samples from cold regions of the estimated field. Percentage values of each axis represent the variation explained.

After our quality analysis, we performed further analysis to find the exact microorganisms present in our samples and examined the differences between hot and cold regions estimated by our framework. Figure 15 shows the principal coordinate analysis (PCoA) (Anderson and Willis, 2003), which is a commonly used method to find the dissimilarities between a group of microorganisms in a sample. Although we performed only three experiments, the results shown in Figure 15 are encouraging. It is clearly evident that the group of microorganisms living in the hot regions are substantially different from the group living in the cold regions of the estimated fields. Therefore, these preliminary results provide a good use case for the adaptive frameworks. Such field estimation experiments can further help in understanding the biological questions such as explaining the difference in the groups of microorganisms between the cold and hot regions.

## 6. Conclusion

We outlined a framework for monitoring scalar environmental fields using a team of robots with bounds on overall mission time. We used the kernel information of the sparse GP model to explore the combinations of actions available to the team of robots and collect informative data. The paths are evaluated to minimize the overall variance and we include the time taken for this evaluation in our overall mission time to provide real-time performance. We simulated the framework using real world data and the results show that our framework is capable of coordinating a team of robots efficiently. We also simulated multiple runs of the framework to test the robustness in our performance and the results show consistent results across multiple simulations.

We designed two robots based on the NUSwan vehicle for monitoring reservoirs in Singapore. Using this team of robots, we validated the performance of our framework in the field against conventional methods such as using lawn mower paths. The estimation error for these field experiments was based on the test data collected after finishing the monitoring task and the results show that our framework outperforms the lawn mower approach. Overall, we explained and validated our contribution for using a team of robots to estimate a scalar environmental field.

We further examined the biological relevance of the fields estimated using our multi-robot framework, *m-AdaPP*. We used the framework to estimate three fields and find the regions of high (hot) and low (cold) concentrations for each survey area. After completing each survey, we collected physical water samples using our robots and used standard scientific protocols to analyze the communities of microorganisms in the samples. These standard lab-based methods were sample filtering, DNA sequencing, and assembling the DNA to identify different microorganisms. The results show the samples collected using our framework are of good quality and can be used for biological studies. Moreover, we analyzed our samples collected from hot and cold regions and found the microorganism communities to be distinct.

## 7. Limitations and Future Work

The suggested m-AdaPP framework has two limitations. First, the centralized approach for coordinating the team of robots. Our framework solves the best actions for the entire team of robots and thus the size of the decision space is directly related to the number of robots. This direct relationship results in high computational cost for a large team of robots. An approach to address this limitation can be a distributed algorithm.

The second limitation comes from the use of SPGP. Although the training time scales with *NM*^2^ instead of *N*^3^ still having a very large number of training points, *N* will affect the performance of our framework. A simple solution to this problem will be the use of streaming GPs as the training time as these GP models are completely independent of the training points *N*.

The field experiments primarily showed the use for in-water applications. However, the problem formulation of our framework does not put a limitation on the applications and it can be easily extended to estimate any scalar field that can be approximated using GPs. Our framework can be easily used for the estimation of air temperature or estimation of vegetation spread using aerial or land vehicles.

## Data Availability Statement

The dataset used in simulations is available publicly and the citations are available in this paper. The data collected via field experiments is confidential.

## Author Contributions

RM contributed in problem formulation, implementation of the framework, simulations, system design, field experiments, and sample collection using the robots. TK contributed in system design and provided supervision for field experiments and sample collection using the robots. MC contributed in problem formulation and provided supervision for implementation of the framework, analysis of simulation results, and system design. SS contributed in managing field experiments, freshwater sample collection as well as its analysis. All authors contributed to the article and approved the submitted version.

## Conflict of Interest

The authors declare that the research was conducted in the absence of any commercial or financial relationships that could be construed as a potential conflict of interest.
